# Breast cancer risk prediction in women aged 35–50 years: impact of including sex hormone concentrations in the Gail model

**DOI:** 10.1186/s13058-019-1126-z

**Published:** 2019-03-19

**Authors:** Tess V. Clendenen, Wenzhen Ge, Karen L. Koenig, Yelena Afanasyeva, Claudia Agnoli, Louise A. Brinton, Farbod Darvishian, Joanne F. Dorgan, A. Heather Eliassen, Roni T. Falk, Göran Hallmans, Susan E. Hankinson, Judith Hoffman-Bolton, Timothy J. Key, Vittorio Krogh, Hazel B. Nichols, Dale P. Sandler, Minouk J. Schoemaker, Patrick M. Sluss, Malin Sund, Anthony J. Swerdlow, Kala Visvanathan, Anne Zeleniuch-Jacquotte, Mengling Liu

**Affiliations:** 1 Department of Population Health, New York University School of Medicine, 650 First Avenue, New York, NY 10016 USA; 2 Department of Pathology, New York University School of Medicine, New York, NY USA; 3 Perlmutter Cancer Center, New York University School of Medicine, New York, NY USA; 4 Epidemiology and Prevention Unit, Fondazione IRCCS - Istituto Nazionale dei Tumori, Milan, Italy; 5 Division of Cancer Epidemiology and Genetics, National Cancer Institute, National Institutes of Health, Bethesda, MD USA; 6 Department of Epidemiology and Public Health, University of Maryland School of Medicine, Baltimore, MD USA; 7 Department of Epidemiology, Harvard T.H. Chan School of Public Health, and Channing Division of Network Medicine, Brigham and Women’s Hospital, Harvard Medical School, Boston, MA USA; 8 Department of Biostatistics and Epidemiology, School of Public Health and Health Sciences, University of Massachusetts, Amherst, MA USA; 9 Department of Biobank Research, Public Health and Clinical Medicine, Umeå University, Umeå, Sweden; 10 Department of Epidemiology, Johns Hopkins Bloomberg School of Public Health, Baltimore, MD USA; 11 Sidney Kimmel Cancer Center, Johns Hopkins School of Medicine, Baltimore, MD USA; 12 Cancer Epidemiology Unit, Nuffield Department of Population Health, University of Oxford, Oxford, UK; 13 Department of Epidemiology, University of North Carolina, Chapel Hill, NC USA; 14 Epidemiology Branch, National Institute of Environmental Health Sciences, Research Triangle Park, NC USA; 15 Division of Genetics and Epidemiology, The Institute of Cancer Research, London, UK; 16 Division of Breast Cancer Research, The Institute of Cancer Research, London, UK; 17 Department of Pathology, Harvard Medical School, Boston, MA USA; 18 Department of Surgery, Umeå University Hospital, Umeå, Sweden

**Keywords:** Breast cancer risk prediction, Anti-Müllerian hormone, Testosterone, Gail model

## Abstract

**Background:**

Models that accurately predict risk of breast cancer are needed to help younger women make decisions about when to begin screening. Premenopausal concentrations of circulating anti-Müllerian hormone (AMH), a biomarker of ovarian reserve, and testosterone have been positively associated with breast cancer risk in prospective studies. We assessed whether adding AMH and/or testosterone to the Gail model improves its prediction performance for women aged 35–50.

**Methods:**

In a nested case-control study including ten prospective cohorts (1762 invasive cases/1890 matched controls) with pre-diagnostic serum/plasma samples, we estimated relative risks (RR) for the biomarkers and Gail risk factors using conditional logistic regression and random-effects meta-analysis. Absolute risk models were developed using these RR estimates, attributable risk fractions calculated using the distributions of the risk factors in the cases from the consortium, and population-based incidence and mortality rates. The area under the receiver operating characteristic curve (AUC) was used to compare the discriminatory accuracy of the models with and without biomarkers.

**Results:**

The AUC for invasive breast cancer including only the Gail risk factor variables was 55.3 (95% CI 53.4, 57.1). The AUC increased moderately with the addition of AMH (AUC 57.6, 95% CI 55.7, 59.5), testosterone (AUC 56.2, 95% CI 54.4, 58.1), or both (AUC 58.1, 95% CI 56.2, 59.9). The largest AUC improvement (4.0) was among women without a family history of breast cancer.

**Conclusions:**

AMH and testosterone moderately increase the discriminatory accuracy of the Gail model among women aged 35–50. We observed the largest AUC increase for women without a family history of breast cancer, the group that would benefit most from improved risk prediction because early screening is already recommended for women with a family history.

**Electronic supplementary material:**

The online version of this article (10.1186/s13058-019-1126-z) contains supplementary material, which is available to authorized users.

## Background

Breast cancer risk prediction models can help women and their health providers make decisions about screening and chemoprevention. While women aged 50 are uniformly included in mammographic screening recommendations, the guidelines regarding at what age to start screening are inconsistent, varying from age 40 to 50, particularly for women without a family history of breast cancer (https://www.uspreventiveservicestaskforce.org/Page/Document/UpdateSummaryFinal/breast-cancer-screening1 [1–7]). Improvements in individualized risk assessment would therefore be particularly valuable for women younger than 50 to decide when to start mammographic screening. A risk prediction model with high accuracy could also help women decide whether to take tamoxifen for breast cancer prevention. Younger women are more likely to benefit from tamoxifen than older women because they have lower risks of tamoxifen-related adverse events [8–13]. Nonetheless, an accurate estimate of risk of breast cancer is critical in calculating the benefit-risk index for these women.

The Gail model 2 [14] is the most widely studied breast cancer risk prediction model for women without a strong family history of breast cancer or an inherited mutation associated with high susceptibility. The breast cancer risk factors in the model are age, age at menarche, age at first live birth, number of previous breast biopsies, history of atypical hyperplasia, and first-degree family history of breast cancer [14]. The Gail model 2 was initially developed using data from white women, and race/ethnicity-specific adaptations of the model were subsequently developed. The model was implemented in the National Cancer Institute’s Breast Cancer Risk Assessment Tool (BCRAT) which is available online. The model has been validated in studies in the USA and several Western European countries, including studies of younger women [15–23]. It has been shown in most studies to be well calibrated [14, 15, 23], i.e., it predicts fairly accurately the number of women who will develop breast cancer overall and in subgroups defined by risk factors. However, the model has limited discriminatory accuracy, i.e., it does not separate well women who subsequently develop cancer from those who do not [15].

We recently showed that the premenopausal circulating concentration of anti-Müllerian hormone (AMH), a marker of ovarian reserve, is associated with risk of breast cancer [24]. Circulating testosterone concentration, measured before [25–30] or after menopause [31–38], has also been consistently associated with breast cancer risk. AMH and testosterone are fairly stable during the menstrual cycle and temporal reliability studies have shown that a single measurement of AMH or testosterone can be used to rank premenopausal women with regard to their average hormone level over a several-year period with reasonable accuracy [25, 34, 39–42]. They are also relatively inexpensive to measure. Thus, these two hormones are good candidate biomarkers for inclusion in breast cancer risk prediction models for younger women, who have large fluctuations in other hormone-related biomarkers during the menstrual cycle.

The objective of this study was to evaluate whether adding circulating AMH and/or testosterone measurements to the Gail model improves its discriminatory accuracy among women aged 35–50.

## Methods

### Study subjects

Participants in a nested case-control study in a consortium of ten prospective cohorts from the USA, UK, Italy, and Sweden [24] were included in this study. The parent cohorts were the Generations Study (BGS); CLUE II; Columbia, MO Serum Bank (CSB); Guernsey Cohort; New York University Women’s Health Study (NYUWHS); Nurses’ Health Studies (NHS) I and II; Northern Sweden Mammary Screening Cohort (NSMSC); Hormones and Diet in the Etiology of Breast Cancer (ORDET); and the Sister Study (Sister). A brief description of the cohorts can be found in Ge et al. [24]. Each cohort was approved by its institutional review board, and informed consent was obtained from each participant.

Incident breast cancer cases were ascertained by each cohort through self-report on follow-up questionnaires and/or linkages with local, regional, or national cancer registries. All cases of incident invasive breast cancer diagnosed among women who were 35–50 at the time of blood donation were included except in the NHS cohorts, which further limited case selection to women who were premenopausal and between the ages of 35–50 at diagnosis. Controls were selected within each cohort using incidence density sampling. One control was selected for each case (except for the Sister Study, which matched 1:2). Matching variables included age and date of blood donation, and race/ethnicity [24]. Many of the cohorts matched on additional variables, for example, phase or day of menstrual cycle and technical sample characteristics, such as time between collection and processing. Women who were ever users of hormone therapy (HT) or current users of oral contraceptives (OCs) were excluded.

### Laboratory measurements

AMH was measured in serum or plasma samples from women who were premenopausal at the time of blood donation using the picoAMH assay (ANSH laboratories) [24]. Women who had AMH concentrations below the lowest detectable value (LDV) (< 10% of samples for eight cohorts and < 20% for the remaining two cohorts) were classified into the lowest quartile for analyses (see “Statistical methods”). Because it has previously been shown that postmenopausal women have AMH concentrations below the LDV [43, 44], we did not measure AMH in postmenopausal women (23 cases and 40 controls) but also classified them into the lowest quartile.

Total testosterone was measured for all subjects in CLUE II, NHS, and NSMSC and for the matched sets for which it was not measured previously for the other cohorts. Measurements were done in the Immunochemical Core Laboratory of the Mayo Clinic by liquid chromatography-tandem mass spectrometry (LC-MS/MS). Assay coefficients of variation (CVs) were calculated using blinded quality control samples. For AMH, the mean intra-batch CV was 5.1% and the inter-batch CV was 21.4%. For testosterone, all intra- and inter-batch CVs were ≤ 10.6%. Previous testosterone measurements were performed as described in [25, 26, 29, 45–48].

### Statistical methods

#### Relative risk estimation

We estimated cohort-specific relative risks (RRs) associated with the breast cancer risk factors included in the Gail model and with each of the biomarkers (testosterone and AMH) using conditional logistic regression (odds ratio estimates are referred to throughout as relative risks (RRs), by convention). Cohort-specific RRs were combined to obtain consortium-wide RR estimates using the random-effects meta-analytic method. *I*^2^ and *Q*-tests were used to test for heterogeneity across cohorts.

We used the same coding as the BCRAT for age at menarche (< 12 years, 12 to 13, or ≥ 14) and age at first live birth (< 20, 20 to 24, 25 to 29/nulliparous, or ≥ 30 years) [14]. Family history of breast cancer was coded using a three-category variable (0/1/> 1 affected relative(s)). For cohorts that collected family history as a yes/no variable, women who responded yes were included in the intermediate category (1 affected relative). History of breast biopsy was coded as yes/no. We did not include an interaction between breast biopsy and age (< 50/≥ 50 years) because this study was restricted to younger women (≤ 50). The interaction term between age at first birth and number of affected relatives was not statistically significant for any cohort and thus not included in the model. To be consistent with BCRAT, which imputes missing data to the lowest risk category, we imputed missing data as follows: age at menarche: ≥ 14 for 35 cases (1.5%) and 49 controls (1.9%); age at first live birth: < 20 for 5 cases (0.2%) and 7 (0.3%) controls; and number of breast biopsies: 0 for 42 cases (1.8%) and 40 controls (1.6%). Data on history of atypical hyperplasia were not available from any of the cohorts and this variable was set to the lowest risk category as is the case when “unknown” is entered in the BCRAT. Because we could not exclude the possibility that cohort differences in the AMH and testosterone concentration distributions were related to collection/handling/storage of samples [24], biomarkers were categorized into quartiles using cohort-specific cutpoints and modeled as ordered categorical variables.

#### Absolute risk estimation

We used the method described by Gail et al. [22, 49] to estimate the 5-year absolute breast cancer risk for each participant. We used consortium-wide estimates of RRs for the Gail variables and biomarkers (calculated as described above), consortium-based estimates of attributable risk fractions, and population-based breast cancer incidence and mortality rates. Attributable risk fractions were estimated using consortium-wide RR estimates and distributions of the Gail variables and biomarkers in the cases (excluding the Sister Study because all women in this study had a family history of breast cancer) [49]. Breast cancer incidence and competing mortality (i.e., non-breast cancer mortality) rates were obtained from the countries of the participating cohorts (US, UK, Italy, and Sweden) for the relevant 5-year age categories (35–39, 40–44, 45–49) and calendar years of blood collection (Additional file 1: Table S1).

For comparison, we also calculated the 5-year absolute risks of developing breast cancer using the BCRAT SAS macro (available at: https://dceg.cancer.gov/tools/risk-assessment/bcrasasmacro), which uses US population-based RR estimates [8, 14, 15, 22]. We refer to results using these calculations as “BCRAT” (to distinguish them from results based on RRs estimated from our dataset, called “Gail model”).

#### Assessment of discriminatory accuracy

We estimated the area under the receiver operating characteristic curve (AUC) based on the 5-year absolute risk estimates from the BCRAT, the Gail model, and the Gail model with addition of AMH and/or testosterone. Summary AUCs were estimated from the cohort-specific AUCs using random-effects meta-analytic methods. AUCs were also estimated within subgroups, i.e., by age, estrogen receptor (ER) status of the tumor, and Gail risk score (< 1%/≥ 1%), and for women without a family history of breast cancer. AUCs are expressed throughout as percentages (AUC × 100) for ease of interpretation. Finally, we assessed reclassification of 5-year absolute risks upon addition of biomarkers.

## Results

Descriptive characteristics of the cases and controls are shown in Table 1. By design, women were between the ages of 35–50 at blood donation. About 40% of cases donated blood samples in the 5 years preceding breast cancer diagnosis. Consistent with known breast cancer risk factor associations, cases were more likely than controls to have had a breast biopsy, to have a family history of breast cancer, and to be nulliparous or have had their first live birth after age 30. The vast majority of women had low to average BCRAT 5-year risk scores (over half of the women had a risk < 1%), as expected in a study of younger women.

**Table 1:** Descriptive characteristics of invasive breast cancer cases and matched controls

	Cases (*n* = 1762)	Controls (*n* = 1890)
Cohort, *n*		
BGS	230	230
CLUE II	87	87
CSB	69	69
Guernsey	124	124
NHS	93	93
NHS II	248	250
NSMSC	31	31
NYUWHS	493	496
ORDET	214	224
Sister	173	286
Age at blood donation, years, *n* (%)		
35–40	472 (26.8)	487 (25.8)
41–45	708 (40.2)	752 (39.8)
46–50^a^	582 (33.0)	651 (34.5)
Race/ethnicity, *n* (%)		
White	1587 (90.1)	1696 (89.7)
Black/African American	76 (4.3)	73 (3.9)
Other or missing	99 (5.6)	121 (6.4)
Age at diagnosis, years, *n* (%)		
35–45	287 (16.3)	
46–50	579 (32.9)	
51–55	436 (24.7)	
56–60	235 (13.3)	
61–65	141 (8.0)	
> 65	84 (4.8)	
Lag time between blood donation and diagnosis, years, *n* (%)		
0–2	274 (15.6)	
3–5	420 (23.8)	
6–10	443 (25.1)	
11–15	286 (16.2)	
16–20	201 (11.4)	
> 20	138 (7.8)	
Age at menarche, years, *n* (%)		
< 12	376 (21.3)	411 (21.7)
12–13	976 (55.4)	1012 (53.5)
≥14 or missing^b^	410 (23.3)	467 (24.7)
Age at first live birth, years, *n* (%)		
< 20 or missing^b^	114 (6.5)	143 (7.6)
20–24	457 (25.9)	521 (27.6)
25–29^c^	473 (26.8)	511 (27.1)
≥ 30	304 (17.3)	307 (16.2)
Nulliparous	414 (23.5)	408 (21.5)
Number of benign breast biopsies, *n* (%)		
0 or missing^b^	1339 (76.0)	1559 (82.5)
≥ 1	423 (24.0)	331 (17.5)
0	1311 (74.4)	1415 (74.9)
1^d^	382 (21.7)	412 (21.8)
> 1^d^	69 (3.9)	63 (3.3)
BMI, kg/m^2^, *n* (%)		
< 25	1097 (59.9)	1124 (62.6)
25–29	420 (24.8)	465 (24.0)
≥ 30	234 (15.4)	289 (13.4)
Missing	11	12
AMH cohort-specific quartiles, *n*(%)		
Q1	365 (20.7)	480 (25.4)
Q2	444 (25.1)	468 (24.8)
Q3	453 (25.7)	468 (24.8)
Q4	500 (28.4)	474 (25.1)
Testosterone cohort-specific quartiles, *n* (%)		
Q1	423 (24.0)	511 (27.0)
Q2	414 (23.5)	464 (24.6)
Q3	452 (25.7)	460 (24.3)
Q4	473 (26.8)	455 (24.1)
BCRAT 5-year risk score (%), *n* (%)^e^		
< 0.6%	296 (16.8)	332 (17.6)
0.6–0.99%	679 (38.5)	765 (40.5)
1–1.66%	525 (29.8)	517 (27.3)
1.67–1.99%	110 (6.2)	130 (6.9)
2–2.99%	115 (6.5)	115 (6.1)
≥ 3%	37 (2.1)	31 (1.6)
ER status, *n* (%)		
ER-positive	1139 (79.8)	
ER-negative	289 (20.2)	
Unknown	334	

Note: Cases and controls were matched 1:1 for all cohorts except for Sister Study which matched 1:2

^a^All cases had age at blood donation ≤ 50, though for 24 sets, matched controls ages were ≤ 51.2 years at blood donation

^b^To be consistent with BCRAT, which imputes missing data to the lowest risk category, we imputed missing data as follows: age at menarche: ≥ 14 for 35 cases (1.5%) and 49 controls (1.9%); age at first live birth: < 20 for 5 cases (0.2%) and 7 (0.3%) controls; number of breast biopsies: 0 for 42 cases (1.8%) and 40 controls (1.6%)

^c^As done in BCRAT, nulliparous and women who were 25–29 at first birth were combined in all models

^d^The number of first-degree family members with breast cancer was coded as 0, 1, or > 1 affected relatives. For cohorts that collected family history as a no/yes variable, “yes” answers were assigned to the intermediate category (1 affected relative)

^e^Calculated using the following variables: race, age at menarche, age at first live birth, number of breast biopsies, and number of first-degree family members with breast cancer, history of atypical hyperplasia was missing for all cohorts and set to “no.” Gail model 2 rates and parameters were used as described in [14]

Table 2 shows the RR estimates for invasive breast cancer associated with Gail model risk factors and biomarkers. The RRs for the Gail model variables did not change appreciably with the addition of biomarkers to the model. When individually added to the Gail model, AMH was associated with a 55% increase in risk and testosterone with a 27% increase in risk for the 4th vs. 1st quartiles; when added together, AMH was associated with a 53%, and testosterone with a 22%, increase. Table 2 also shows the attributable risk fraction estimates for each unit increase in risk factor or biomarker. For Gail model variables, the risk attributable to age at menarche was low (< 1%), while attributable risks were higher for family history of breast cancer (7%), history of breast biopsy (8%), and age at first pregnancy (18%). The attributable risk for a one-quartile increase in AMH was 19% and for testosterone 9%. In a sensitivity analysis restricted to the five US cohorts included in our study, the attributable risks calculated using US population risk factor distributions were similar to estimates based on risk factor distributions in the cases (data not shown) [22, 49–51]. Cohort-specific RR estimates for invasive breast cancer from the model including both biomarkers are shown in Additional file 1: Figure S1. Tests for heterogeneity by cohort were not statistically significant. Removing one cohort at a time from the analysis did not change the RRs appreciably (data not shown).

**Table 2:** Relative risks calculated using random-effects meta-analysis and attributable risk fractions

Risk factor	RR estimates				Attributable risk (%) for Gail+ AMH + testosterone model^b^
	Gail	Gail + AMH	Gail + testosterone	Gail + AMH + testosterone	
Age at menarche, years					0.67%
< 12	1.00 (0.90, 1.11)	1.02 (0.91, 1.13)	1.00 (0.90, 1.11)	1.01 (0.91, 1.12)	
12–13	1.00 (0.90, 1.11)	1.01 (0.91, 1.12)	1.00 (0.90, 1.11)	1.01 (0.91, 1.12)	
≥ 14	1.0 (ref)	1.0 (ref)	1.0 (ref)	1.0 (ref)	
Age at first live birth, years					18.47%
< 20	1.0 (ref)	1.0 (ref)	1.0 (ref)	1.0 (ref)	
20–24	1.11 (1.00, 1.24)	1.12 (1.00, 1.25)	1.12 (1.00, 1.26)	1.12 (1.00, 1.26)	
25–29 or nulliparous	1.24 (1.11, 1.38)	1.25 (1.12, 1.39)	1.26 (1.12, 1.41)	1.26 (1.13, 1.42)	
≥ 30	1.38 (1.23, 1.54)	1.40 (1.25, 1.56)	1.41 (1.26, 1.58)	1.42 (1.27, 1.60)	
Number of benign breast biopsies					8.13%
0	1.0 (ref)	1.0 (ref)	1.0 (ref)	1.0 (ref)	
≥ 1	1.58 (1.33, 1.88)	1.55 (1.31, 1.85)	1.59 (1.34, 1.89)	1.56 (1.31, 1.86)	
Number of first-degree family members with breast cancer^a^					6.56%
0	1.0 (ref)	1.0 (ref)	1.0 (ref)	1.0 (ref)	
1	1.58 (1.32, 1.89)	1.57 (1.31, 1.88)	1.57 (1.30, 1.88)	1.56 (1.30, 1.87)	
> 1	2.49 (2.08, 2.99)	2.47 (2.06, 2.96)	2.45 (2.04, 2.94)	2.43 (2.03, 2.92)	
AMH					19.38%
Q1	–	1.0 (ref)	–	1.0 (ref)	
Q2	–	1.16 (1.04, 1.29)	–	1.15 (1.03, 1.28)	
Q3	–	1.34 (1.20, 1.49)	–	1.33 (1.19, 1.48)	
Q4	–	1.55 (1.39, 1.73)	–	1.53 (1.37, 1.70)	
Testosterone					9.48%
Q1	–	–	1.0 (ref)	1.0 (ref)	
Q2	–	–	1.08 (1.02, 1.15)	1.07 (1.00, 1.14)	
Q3	–	–	1.17 (1.10, 1.25)	1.14 (1.07, 1.22)	
Q4	–	–	1.27 (1.19, 1.35)	1.22 (1.15, 1.30)	

^a^The number of first-degree family members with breast cancer was coded as either 0, 1, or > 1 affected relatives. For cohorts that collected family history as a no/yes variable, “yes” answers were assigned to the intermediate category (1 affected relative)

^b^We used the method described in Bruzzi et al. [49] to estimate attributable risk for a one-category increase (or decrease for age at menarche) in the risk factor. The Sister study was excluded from attributable risk estimation because all participants had a family history of breast cancer

**Fig. 1:**
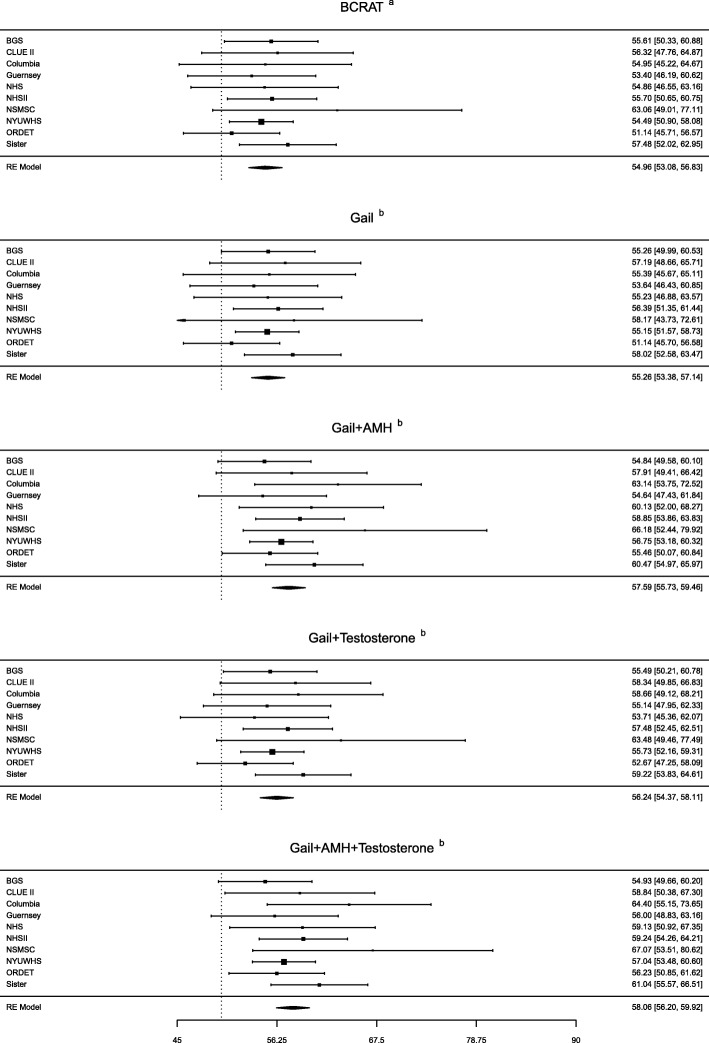
Area under the receiver operating curve (AUC) estimates and 95% confidence intervals

Figure 1 and Table 3 show the AUCs based on BCRAT, the Gail model, and the Gail model with biomarkers. The summary AUC for invasive breast cancer using the BCRAT was 55.0 (95% CI 53.1, 56.8). The AUC in our implementation of the Gail model was very similar (AUC 55.3, 95% CI 53.4, 57.1). The AUC increased with the addition of AMH (AUC 57.6, 95% CI 55.7, 59.5), testosterone (AUC 56.2, 95% CI 54.4, 58.1), and both AMH and testosterone (AUC 58.1, 95% CI 56.2, 59.9). The percent increase relative to the Gail model was statistically significant for the model including AMH (4.2%, *p* = 0.007) and the model including both AMH and testosterone (5.1%, *p* = 0.001), but not testosterone alone (1.6%, *p* = 0.086). AUCs were similar when both in situ and invasive cases were considered together (Additional file 1: Figure S4).

**Table 3:** AUCs by subgroups

	BCRAT^a^	Gail^b^	Gail + AMH^b^	Gail + testosterone^b^	Gail + AMH + testosterone^b^
Total AUC	55.0 (53.1, 56.8)	55.3 (53.4, 57.1)	57.6 (55.7, 59.5)	56.2 (54.4, 58.1)	58.1 (56.2, 59.9)
Age at blood donation, years					
≤ 40	55.9 (52.3, 59.6)	56.2 (52.5, 59.8)	57.5 (53.8, 61.1)	57.3 (53.7, 61.0)	58.1 (54.4, 61.8)
41–45	55.2 (52.2, 58.2)	54.9 (51.9, 57.9)	56.3 (53.3, 59.2)	56.0 (53.0, 58.9)	56.6 (53.7, 59.6)
> 45	58.6 (55.4, 61.9)	58.6 (55.3, 61.9)	60.6 (57.4, 63.8)	60.9 (57.7, 64.1)	62.1 (58.9, 65.3)
Gail 5-year risk score, %					
< 1^c^	53.2 (50.2, 55.2)	52.9 (50.4, 55.4)	54.7 (52.2, 57.2)	54.3 (51.8, 56.8)	55.9 (53.4, 58.3)
≥ 1^c^	56.6 (53.7, 59.5)	58.2 (55.3, 61.0)	59.1 (56.3, 62.0)	57.4 (54.3, 60.5)	59.2 (56.3, 62.1)
Estrogen receptor status					
ER-positive	56.1 (53.8, 58.4)	56.4 (54.1, 58.8)	58.9 (56.2, 61.6)	57.2 (54.9, 59.5)	59.2 (56.3, 62.0)
ER-negative	55.8 (51.1, 60.5)	56.8 (52.1, 61.5)	58.0 (53.3, 62.7)	57.1 (52.4, 61.8)	57.1 (52.3, 61.8)
Number of first-degree family members with breast cancer, *n* (%)					
0	52.2 (50.0, 54.3)	52.8 (50.6, 55.0)	55.6 (52.9, 58.3)	54.6 (52.4, 56.8)	56.8 (54.6, 58.9)
≥ 1	55.9 (52.1, 59.6)	55.0 (51.3, 58.7)	57.2 (53.4, 60.9)	56.4 (52.7, 60.1)	57.2 (52.0, 62.4)

^a^Estimates from the model as implemented in BCRAT and using BCRAT regression coefficients

^b^Model including Gail model variables and biomarker(s) and using regression coefficients in Table 2

^c^Median 5-year absolute risk was approximately 1%

Table 3 also shows AUCs in subgroups. Small improvements in AUCs with the addition of both biomarkers to the Gail model were observed in each age-at-blood-donation subgroup, with the largest increase (3.5, a relative increase of 6.0%) for women ages 45–50, for whom the Gail model also had the highest AUC (58.6). AUC improvements for women with a 5-year risk lower than 1% were greater (3.0, a relative increase of 5.7%) than those for women with risk of at least 1% (1.0, a relative increase of 1.7%). AUC improvement was larger for ER-positive tumors (2.8, a relative increase of 5.0%) than ER-negative tumors (0.3, a relative increase of 0.5%). We also found that the AUC increased (4.0, a relative increase of 7.6%) with the addition of biomarkers for the subgroup of women without a family history of breast cancer, but less so for women with a family history (2.2, a relative increase of 4.4%).

**Fig. 2:**
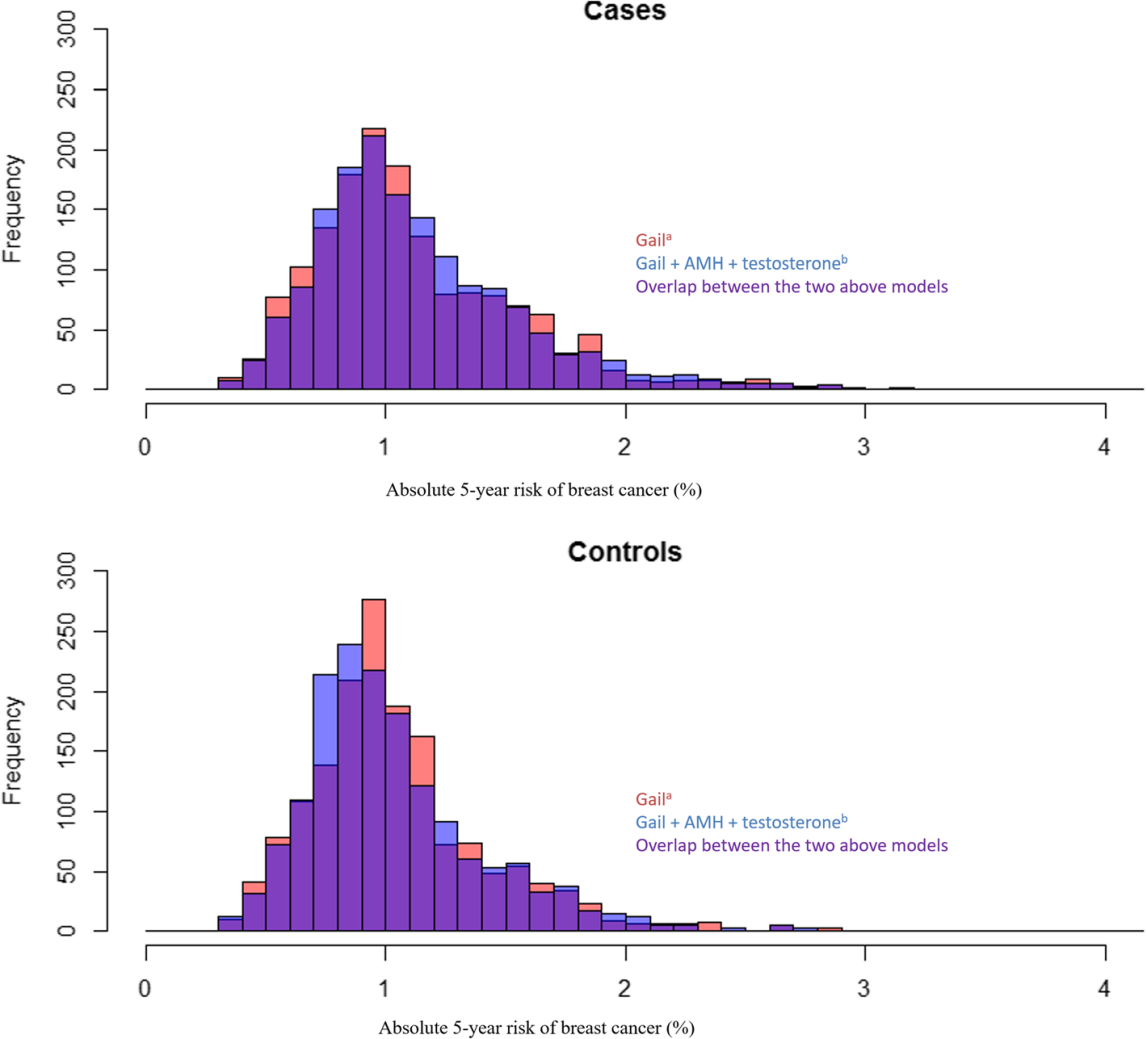
Reclassification of absolute 5-year risk of breast cancer with the addition of AMH and testosterone to the Gail model

Figure 2 shows the histograms displaying absolute risk estimates of cases and controls for the Gail model with and without testosterone and AMH. Though there was substantial overlap between the distributions in cases and controls, the distribution was skewed to the right for cases. Adding the biomarkers resulted in a slight shift of the distribution to the right for cases (9.3% had risk estimates move from below to above 1%, while 8.1% moved down, Table 4) and a slight shift to the left for controls (8.7% had risk estimates move from below to above 1%, while 10.4% moved down, Table 4).

**Table 4:** Absolute risk reclassification upon adding AMH and testosterone to the Gail model

		Gail + AMH + testosterone 5-year risk		Moved up (%)	Moved down (%)
Reclassification in cases		< 1%	≥ 1%		
Gail 5-year risk	< 1%	588	163		
	≥ 1%	143	868		
				9.3%	8.1%
Reclassification in controls		< 1%	≥ 1%		
Gail 5-year risk	< 1%	708	165		
	≥ 1%	196	821		
				8.7%	10.4%

## Discussion

Circulating AMH and testosterone moderately increased the discriminatory accuracy of the Gail breast cancer risk prediction model among women ages 35–50 in our study of 1762 invasive cases and 1890 matched controls. Discriminatory accuracy improved with the addition of either AMH or testosterone, though the improvement was only statistically significant for AMH. In the model including both biomarkers, we observed an AUC increase from 55.3 to 58.1 (relative increase of 5.1%). Overall, inclusion of biomarkers tended to moderately increase 5-year risk estimates for cases and reduce estimates for controls.

The increase in AUC resulting from the addition of biomarkers was slightly higher in analyses limited to women without a family history of breast cancer than that observed in analyses including all women. This is of interest because the majority of breast cancers occur among women without a family history. Further, women without a family history are the group in which improvements in risk prediction could have the most impact, since it is already recommended that women with a family history start screening early (https://www.uspreventiveservicestaskforce.org/Page/Document/UpdateSummaryFinal/breast-cancer-screening1).

While risk prediction models applicable to younger women would be valuable for screening and preventive treatment decision-making, less work has focused on this group of women as compared to older women [52–54]. To our knowledge, risk prediction estimation has been assessed for premenopausal women from the general population in six studies [55–60]. Most of these assessed or modified the Gail model, but some had extensive missing data for Gail model variables [55, 57] or did not assess discriminatory accuracy [57]. Others developed new models for which validation has not yet been attempted in independent studies [55, 60]. Testosterone was added to the Gail model in one study that included premenopausal women [56]. In this study of 430 cases/684 controls, the addition of hormones, including testosterone, to the Gail model did not result in any change in the AUC for premenopausal women [56]. Unlike this study, the increase in AUC that we observed with the addition of testosterone is in agreement with the premenopausal testosterone-breast cancer risk association that has been consistently observed [25–30]. AMH has not been included in breast cancer risk prediction models previously.

Some studies, though not all [61, 62], have reported correlations of BMI with testosterone and AMH in premenopausal women [39, 63, 64]. These correlations have generally been weak, including in our study (Spearman partial correlations with BMI among controls, adjusted for cohort and age, were 0.06 for testosterone, and − 0.07 for AMH). This suggests that including BMI in the model, though it would be easier than including biomarkers because BMI does not require a blood draw, would not capture the impact of AMH and testosterone on breast cancer risk.

The AUC increases with the addition of AMH, and testosterone were greater for ER-positive than ER-negative tumors, as expected since AMH was more strongly associated with risk of ER-positive than ER-negative tumors in our study [24]. Though AMH and estrogen concentrations are not strongly correlated in premenopausal women [39, 64], AMH is strongly associated with age at menopause, at which time estrogen exposure decreases. This association may explain the greater improvement in prediction of estrogen-sensitive tumors than ER-negative tumors with the inclusion of AMH in the Gail model.

Several other risk factors have been proposed for inclusion in the Gail model to improve discriminatory accuracy, with varying applicability to premenopausal women. Mammographic density has been shown to increase the discriminatory accuracy of the Gail model in several studies [51, 55, 65, 66], but density is not available yet to women deciding when to begin screening. Endogenous hormones other than AMH and testosterone, such as estrogen, progesterone, and prolactin, fluctuate during the menstrual cycle and/or are not consistently associated with risk in premenopausal women [31, 67]. Common, low-penetrance genetic risk factors may also have utility for risk prediction in younger women. Single nucleotide polymorphisms (SNPs), and their combined risk scores (ranging from 6 to 77 SNPs across studies), have increased Gail model AUCs (AUC increases of 0.6–7.0) in most studies [54, 59, 68–75], including among younger women [59]. Inclusion of a 77-SNP score increased the AUC from 0.64 to 0.66 among women < 50 years of age [59], an increase comparable to that observed with the addition of AMH and testosterone. Because most genetic variants that are associated with breast cancer risk are not in hormone-related genes, they are likely to contribute to risk prediction independently of AMH and testosterone. Thus, models including both genetic variants and hormone biomarkers as a panel may perform better than models including only one type of marker.

We could not directly assess the calibration of the model including biomarkers because AMH and testosterone were measured only in matched case-control sets; thus, the expected number of cases in the full cohorts using the model including biomarkers could not be estimated [76]. Another method to indirectly assess calibration is inverse probability weighting [77], which uses the probability of being selected into the nested case-control study as a weighting factor to estimate the expected number of cases in the cohort. However, closely matched nested case-control studies, as in this consortium, yield high selection probabilities for a substantial proportion of controls because the risk sets from which controls are selected can be very small. For example, for the 496 controls in the NYUWHS, we would expect an average selection probability of ~ 10% (5600 cohort participants were between the ages of 35 and 50 at enrollment), but the average probability was 35%. The controls in this study provided insufficient information about the full cohort, precluding the assessment of calibration [76].

Our study included past users of oral contraceptives (> 65%) [24], but not current users because AMH levels go down during oral contraceptive use [62, 78, 79]. Thus, our results only apply to women not on oral contraceptives.

In addition to the large size of our study, its major strength is the prospective design. Samples collected prior to diagnosis are valuable for measuring biomarkers that can be affected by the diagnosis and/or treatment of breast cancer. Another strength is that detailed epidemiological data on breast cancer risk factors were collected from all cohorts.

## Conclusions

In conclusion, we observed moderate increases in the discriminatory accuracy of the Gail model 2 for women aged 35–50 with the addition of AMH and testosterone. Combining these markers with others (e.g., SNPs) may improve risk prediction models, though the improvement in discriminatory accuracy will remain limited until new markers with stronger associations with breast cancer risk are identified [80, 81].

## Additional file

Additional file 1: **Table S1.** Breast cancer incidence and competing mortality rates used for each cohort to estimate absolute risk. **Table S2.** Descriptive characteristics of invasive + in situ cases and matched controls. **Table S3.** Descriptive characteristics of invasive breast cancer cases and matched controls, by cohort. **Table S4.** Descriptive characteristics of invasive plus in situ breast cancer cases and matched controls, by cohort. **Table S5.** Random-effects meta-analysis relative risk estimates, invasive and in situ. **Figure S1.** Cohort-specific and random-effects meta-analysis relative risk estimates for Gail model variables, AMH and testosterone (invasive cases only). **Figure S2.** Cohort-specific and random-effects meta-analysis relative risk estimates for Gail model variables, AMH and testosterone, invasive and in situ. **Figure S3.** Relative risk estimates by age group, invasive cases only. **Figure S4.** AUCs by cohort 95% confidence intervals, invasive and in situ. (DOCX 254 kb)

## Abbreviations

AMH: Anti-Müllerian hormone
AUC: Area under the receiver operating characteristic curve
BCRAT: Breast Cancer Risk Assessment Tool
BGS: The Generations Study
BMI: Body mass index
CSB: Columbia, MO Serum Bank
CV: Coefficient of variation
ER: Estrogen receptor
LDV: Lowest detected value
NHS: Nurses’ Health Study
NSMSC: Northern Sweden Mammary Screening Cohort
NYUWHS: New York University Women’s Health Study
ORDET: Hormones and Diet in the Etiology of Breast Cancer
RR: Relative risk
