# Supplementary material for: Breast cancer risk prediction in women aged 35–50 years: impact of including sex hormone concentrations in the Gail model
Source: Breast Cancer Res. 2019 Mar 19;21:42. doi: 10.1186/s13058-019-1126-z (PMC6425605; doi:10.1186/s13058-019-1126-z)
Supplement: Supplementary file 1 — Table S1. Breast cancer incidence and competing mortality rates used for each cohort to estimate absolute risk. Table S2. Descriptive characteristics of invasive + in situ cases and matched controls. Table S3. Descriptive characteristics of invasive breast cancer cases and matched controls, by cohort. Table S4. Descriptive characteristics of invasive plus in situ breast cancer cases and matched controls, by cohort. Table S5. Random-effects meta-analysis relative risk estimates, invasive and in situ. Figure S1. Cohort-specific and random-effects meta-analysis relative risk estimates for Gail model variables, AMH and testosterone (invasive cases only). Figure S2. Cohort-specific and random-effects meta-analysis relative risk estimates for Gail model variables, AMH and testosterone, invasive and in situ. Figure S3. Relative risk estimates by age group, invasive cases only. Figure S4. AUCs by cohort 95% confidence intervals, invasive and in situ. (DOCX 254 kb) [file 13058_2019_1126_MOESM1_ESM.docx]

Supplementary methods

Laboratory methods

Total testosterone was measured for all subjects in CLUE II, NHS, and NSMSC, and for the matched sets for which it was not measured previously for the other cohorts. Measurements for this study were done in the Immunochemical Core Laboratory of the Mayo Clinic by liquid chromatography-tandem mass spectrometry (LC-MS/MS) (ThermoFisher Scientific, Franklin, MA and Applied Biosystems-MDS Sciex, Foster City, CA). Previous testosterone measurements were performed as described in [1-7].

Samples from each cohort were analyzed together and matched sets were analyzed in the same batch. For AMH measurements and testosterone newly measured for this study, each batch included blinded pooled quality control samples from both a consortium-wide pool (~5% per batch) and a cohort-specific pool (~5% per batch).

For the AMH measurements, QCs had a mean intra-batch CV of 5.1% and the inter-batch CV was 21.4%. Intra- and inter-batch CVs for testosterone were ≤10.6%.

We performed repeat AMH measurements in the Ansh laboratory on a subset of 30 samples each from the two cohorts (NYUWHS and Sister Study) for which measurements had originally been done in different laboratories and observed intra-class correlations (ICCs) >98% with measurements from the original laboratories [8]. Samples that had been used to perform testosterone measurements in previous studies (i.e., not newly measured) were available for only a subset of the cohorts. ICCs for previous vs. new testosterone measurements for a ~30 samples per cohort ranged from 0.77-0.95 for BGS, CSB, NYUWHS, ORDET, and Sister.

Raw AMH and testosterone measurements were used to define quartile cutpoints for all analyses. For cohorts with both old and new testosterone measurements, cutoffs were defined by batch-specific quartiles in controls. Analyses based on raw data were not appreciably different from those using data calibrated with regression equations (i.e., previous measurements calibrated to Ansh laboratory for AMH and to Mayo laboratory for testosterone) for the subset of cohorts that had calibration sample measurements.

Sensitivity analysis of attributable risk estimation in US cohorts

For the US cohorts, we also calculated attributable risks using population-based distributions of the Gail-model risk factors. Data from the US National Health Interview Survey collected during the enrollment years of each US cohort from women ages 35-50 were used for this analysis. Estimates of risk attributable to each of the Gail model variables calculated using these US population data were similar to estimates based on risk factor distributions from our US cohorts (data not shown)[9-12].

Table S1: Breast cancer incidence and competing mortality rates used for each cohort to estimate absolute risk

| Cohort | Country | Years of blood donation | Incidence rates | Competing (non-breast cancer) mortality rates |
| --- | --- | --- | --- | --- |
| BGS | UK | 2003-2010 | UK, England, 2003-2007 | UK, 2005-2010 |
| CLUE II | USA | 1989 | US SEER, 1988-1992 | US, 1990-1995 |
| CSB | USA | 1977-1987 | US SEER, 1983-1987 | US, 1985-1990 |
| Guernsey | UK | 1977-1990 | UK, England and Wales 1983-1987 | UK, 1985-1990 |
| NHS | USA | 1989-1990 | US SEER, 1988-1992 | US, 1990-1995 |
| NHSII | USA | 1996-1999 | US SEER, 1998-2002 | US, 2000-2005 |
| NSMSC | Sweden | 1995-2006 | Sweden, 1998-2002 | Sweden, 2000-2005 |
| NYUWHS | USA | 1985-1991 | US SEER, 1988-1992 | US, 1990-1995 |
| ORDET | Italy | 1987-1992 | Italy, Varese, 1988-1992 | Italy, 1990-1995 |
| Sister Study | USA | 2003-2009 | SEER, 2003-2007 | US, 2005-2010 |

Note: Data on incidence rates from CI5 [13], Breast cancer mortality rates and competing mortality rates from the WHO mortality database [12, 14, 15]

| Table S2: Descriptive characteristics of invasive + in situ cases and matched controls | | |  |  |
| --- | --- | --- | --- | --- |
|  | **Invasive + In Situ Sets** | |  |  |
|  | **Cases Controls**  **(n=2232) (n=2416)** | |  |  |
| Cohort, n |  | |  |  |
| BGS | 268 268 | |  |  |
| CLUE II | 98 98 | |  |  |
| CSB | 77 77 | |  |  |
| Guernsey | 148 148 | |  |  |
| NHS | 118 118 | |  |  |
| NHS II | 356 358 | |  |  |
| NSMSC | 31 31 | |  |  |
| NYUWHS | 658 661 | |  |  |
| ORDET | 231 241 | |  |  |
| Sister | 247 416 | |  |  |
| Age at blood donation, yrs, n (%) |  | |  |  |
| 35 - 40 | 599 (26.8) 625 (25.9) | |  |  |
| 41 - 45 | 893 (40.0) 960 (39.7) | |  |  |
| 46 - 50 ^a^ | 740 (33.2) 831 (34.4) | |  |  |
| Race/ethnicity, n (%) |  | |  |  |
| White | 1997 (89.5) 2242 (88.7) | |  |  |
| Black/African American | 99 (4.4) 106 (4.4) | |  |  |
| Other or missing | 136 (6.1) 168 (7.0) | |  |  |
| Age at diagnosis, yrs, n (%) |  | |  |  |
| 35 - 45 | 366 (16.4) | |  |  |
| 46 - 50 | 725 (32.5) | |  |  |
| 51 - 55 | 549 (24.6) | |  |  |
| 56 - 60 | 302 (13.5) | |  |  |
| 61 - 65 | 180 (8.1) | |  |  |
| >65 | 110 (4.9) | |  |  |
| Lag time between blood donation and diagnosis, yrs, n (%) |  | |  |  |
| 0 - 2 | 340 (15.2) | |  |  |
| 3 - 5 | 535 (24.0) | |  |  |
| 6 - 10 | 556 (24.9) | |  |  |
| 11 - 15 | 364 (16.3) | |  |  |
| 16 - 20 | 260 (11.6) | |  |  |
| >20 | 177 (7.9) | |  |  |
| Age at menarche, yrs, n(%) |  | |  |  |
| <12 | 499 (22.4) 575 (23.8) | |  |  |
| 12 - 13 | 1239 (55.5) 1312 (54.3) | |  |  |
| ≥14 or missing^b^ | 494 (22.1) 529 (21.9) | |  |  |
| Age at first live birth, yrs, n(%) |  | |  |  |
| <20 or missing^b^ | 136 (6.1) 184 (7.6) | |  |  |
| 20 - 24 | 557 (25.0) 656 (27.2) | |  |  |
| 25 - 29 or nulliparous women^c^ | 1162 (52.1) 1181 (48.9) | |  |  |
| ≥ 30 | 377 (16.9) 395 (16.3) | |  |  |
| Number of benign breast biopsies, n(%) |  | |  |  |
| 0 or missing^b^ | 1686 (75.5) 1996 (82.6) | |  |  |
| ≥1 | 546 (24.5) 420 (17.4) | |  |  |
| Number of first degree family members with breast cancer, n(%) | |  | |  |
| 0 | 1613 (72.3) 1765 (73.1) | |  |  |
| 1^d^ | 520 (23.3) 555 (23.0) | |  |  |
| >1^d^ | 99 (4.4) 96 (4.0) | |  |  |
| BMI, kg/m^2^, n(%) |  | |  |  |
| <25 | 1413 (63.7) 1432 (59.7) | |  |  |
| 25 - 29 | 527 (23.8) 596 (24.8) | |  |  |
| ≥30 | 278 (12.5) 371 (15.5) | |  |  |
| Missing | 14 17 | |  |  |
| AMH cohort-specific quartiles, n(%) |  | |  |  |
| Q1 | 470 (21.1) 611 (25.3) | |  |  |
| Q2 | 540 (24.2) 601 (24.9) | |  |  |
| Q3 | 578 (25.9) 600 (24.8) | |  |  |
| Q4 | 644 (28.8) 604 (25.0) | |  |  |
| Testosterone cohort-specific quartiles, n(%) |  | |  |  |
| Q1 | 562 (25.2) 656 (27.2) | |  |  |
| Q2 | 517 (23.2) 609 (25.2) | |  |  |
| Q3 | 560 (25.1) 565 (23.4) | |  |  |
| Q4 | 593 (26.5) 586 (24.3) | |  |  |
| BCRAT 5-year risk score, %, n (%) ^e^ |  | |  |  |
| <0.6%  0.6 - 0.99% | 362 (16.2) 422 (17.5)  844 (37.8) 959 (39.7) | |  |  |
| 1 - 1.66% | 654 (29.3) 664 (27.5) | |  |  |
| 1.67 - 1.99% | 163 (7.3) 168 (7.0) | |  |  |
| 2 - 2.99% | 154 (6.9) 155 (6.4) | |  |  |
| ≥3% | 55 (2.5) 48 (2.0) | |  |  |
| ER status, n(%) |  | |  |  |
| ER-positive | 1312 (79.3) | |  |  |
| ER-negative | 342 (20.7) | |  |  |
| Unknown | 578 | |  |  |

Note: Cases and controls were matched 1:1 for all cohorts except for Sister Study which matched 1:2.

^a^ All cases had age at blood donation ≤50, though matched controls ages were ≤51.2 years at blood donation.

^b^ To be consistent with BCRAT we imputed missing data to the lowest risk category.

^c^ As done in BCRAT, nulliparous and women who were 25-29 at first birth were combined in all models.

^d^ The number of first degree family members with breast cancer was coded as 0 , 1 , or > 1 affected relatives. For cohorts that collected family history as a no/yes variable, “yes” answers were assigned to the intermediate category (1 affected relative).

^e^ Calculated using the following variables: race, age at menarche, age at first live birth, number of breast biopsies, and number of first degree family members with breast cancer; history of atypical hyperplasia was missing for all cohorts and set to “no”. Gail model 2 rates and parameters were used as described in [16].

Table S3: Descriptive characteristics of invasive breast cancer cases and matched controls, by cohort

|  | BGS | | | | Clue II | | | | CSB | | | | Guernsey | | | | NHS | | | | NHS II | | | | NSMSC | | | | NYUWHS | | | | ORDET | | | | Sister | | | |
| --- | --- | --- | --- | --- | --- | --- | --- | --- | --- | --- | --- | --- | --- | --- | --- | --- | --- | --- | --- | --- | --- | --- | --- | --- | --- | --- | --- | --- | --- | --- | --- | --- | --- | --- | --- | --- | --- | --- | --- | --- |
|  | Cases | | Controls | | Cases | | Controls | | Cases | | Controls | | Cases | | Controls | | Cases | | Controls | | Cases | | Controls | | Cases | | Controls | | Cases | | Controls | | Cases | | Controls | | Cases | | Controls | |
|  | 230 | | 230 | | 87 | | 87 | | 69 | | 69 | | 124 | | 124 | | 93 | | 93 | | 248 | | 250 | | 31 | | 31 | | 493 | | 496 | | 214 | | 224 | | 173 | | 286 | |
| Age at blood donation, yrs, n (%) |  |  |  |  |  |  |  |  |  |  |  |  |  |  |  |  |  |  |  |  |  |  |  |  |  |  |  |  |  |  |  |  |  |  |  |  |  |  |  |  |
| 35 - 40 |  | 17.4 |  | 20.0 |  | 33.3 |  | 31.0 |  | 17.4 |  | 18.8 |  | 49.2 |  | 45.2 |  | - |  | - |  | 21.8 |  | 18.4 |  | 3.2 |  | 3.2 |  | 23.9 |  | 24.0 |  | 27.1 |  | 25.6 |  | 8.1 |  | 8.4 |
| 41 - 45 |  | 42.6 |  | 40.9 |  | 34.5 |  | 33.3 |  | 47.8 |  | 44.9 |  | 33.1 |  | 35.5 |  | 30.1 |  | 30.1 |  | 42.7 |  | 49.6 |  | 19.4 |  | 22.6 |  | 39.6 |  | 38.9 |  | 37.9 |  | 34.8 |  | 30.6 |  | 30.1 |
| 46 - 50 ^a^ |  | 40.0 |  | 39.1 |  | 32.2 |  | 35.6 |  | 34.8 |  | 36.1 |  | 17.7 |  | 19.3 |  | 69.9 |  | 69.9 |  | 35.5 |  | 31.6 |  | 77.4 |  | 74.2 |  | 36.5 |  | 37.1 |  | 35.0 |  | 40.6 |  | 61.3 |  | 61.5 |
| Race/ethnicity, n (%) |  |  |  |  |  |  |  |  |  |  |  |  |  |  |  |  |  |  |  |  |  |  |  |  |  |  |  |  |  |  |  |  |  |  |  |  |  |  |  |  |
| White |  | 100 |  | 100 |  | 100 |  | 100 |  | 98.6 |  | 100 |  | 100 |  | 100 |  | 98.9 |  | 98.9 |  | 98.4 |  | 98.8 |  | 100 |  | 100 |  | 71.2 |  | 67.7 |  | 100 |  | 100 |  | 84.4 |  | 89.5 |
| Black/African American |  | - |  | - |  | - |  | - |  | 1.4 |  | - |  | - |  | - |  | 1.1 |  | 1.1 |  | 0.8 |  | 0.8 |  | - |  | - |  | 12.2 |  | 10.7 |  | - |  | - |  | 6.9 |  | 5.9 |
| Other or missing |  | - |  | - |  | - |  | - |  | - |  | - |  | - |  | - |  | - |  | - |  | 0.8 |  | 0.4 |  | - |  | - |  | 16.6 |  | 21.6 |  | - |  | - |  | 8.7 |  | 4.5 |
| Age at diagnosis, yrs, n (%) |  |  |  |  |  |  |  |  |  |  |  |  |  |  |  |  |  |  |  |  |  |  |  |  |  |  |  |  |  |  |  |  |  |  |  |  |  |  |  |  |
| 35 - 45 |  | 29.1 |  |  |  | 8.0 |  |  |  | 8.7 |  |  |  | 11.3 |  |  |  | 1.1 |  |  |  | 23.4 |  |  |  | 9.7 |  |  |  | 7.1 |  |  |  | 11.7 |  |  |  | 18.5 |  |  |
| 46 - 50 |  | 48.3 |  |  |  | 17.2 |  |  |  | 10.1 |  |  |  | 13.7 |  |  |  | 32.3 |  |  |  | 37.1 |  |  |  | 25.8 |  |  |  | 17.2 |  |  |  | 26.6 |  |  |  | 50.9 |  |  |
| 51 - 55 |  | 22.2 |  |  |  | 26.4 |  |  |  | 18.8 |  |  |  | 21.8 |  |  |  | 47.3 |  |  |  | 36.7 |  |  |  | 45.2 |  |  |  | 23.5 |  |  |  | 30.4 |  |  |  | 30.6 |  |  |
| 56 - 60 |  | 0.4 |  |  |  | 26.4 |  |  |  | 20.3 |  |  |  | 19.4 |  |  |  | 19.4 |  |  |  | 2.4 |  |  |  | 9.7 |  |  |  | 23.9 |  |  |  | 22.0 |  |  |  | - |  |  |
| 61 - 65 |  | - |  |  |  | 17.2 |  |  |  | 26.1 |  |  |  | 12.9 |  |  |  | - |  |  |  | 0.4 |  |  |  | 9.7 |  |  |  | 15.2 |  |  |  | 7.9 |  |  |  | - |  |  |
| >65 |  | - |  |  |  | 4.6 |  |  |  | 16.0 |  |  |  | 21.0 |  |  |  | - |  |  |  | - |  |  |  | - |  |  |  | 13.0 |  |  |  | 1.4 |  |  |  | - |  |  |
| Lag time between blood donation and diagnosis, yrs, n (%) |  |  |  |  |  |  |  |  |  |  |  |  |  |  |  |  |  |  |  |  |  |  |  |  |  |  |  |  |  |  |  |  |  |  |  |  |  |  |  |  |
| 0 - 2 |  | 32.2 |  |  |  | 8.0 |  |  |  | 10.1 |  |  |  | 4.0 |  |  |  | 10.8 |  |  |  | 21.8 |  |  |  | 25.8 |  |  |  | 4.5 |  |  |  | 10.3 |  |  |  | 37.6 |  |  |
| 3 - 5 |  | 50.9 |  |  |  | 8.0 |  |  |  | 7.2 |  |  |  | 8.9 |  |  |  | 43.0 |  |  |  | 29.8 |  |  |  | 25.8 |  |  |  | 9.3 |  |  |  | 13.1 |  |  |  | 48.6 |  |  |
| 6 - 10 |  | 17.0 |  |  |  | 26.4 |  |  |  | 10.1 |  |  |  | 16.1 |  |  |  | 34.4 |  |  |  | 38.7 |  |  |  | 38.7 |  |  |  | 24.9 |  |  |  | 31.3 |  |  |  | 13.9 |  |  |
| 11 - 15 |  | - |  |  |  | 23.0 |  |  |  | 24.6 |  |  |  | 16.1 |  |  |  | 11.8 |  |  |  | 9.7 |  |  |  | 9.7 |  |  |  | 25.4 |  |  |  | 30.8 |  |  |  | - |  |  |
| 16 - 20 |  | - |  |  |  | 20.7 |  |  |  | 31.9 |  |  |  | 21.8 |  |  |  | - |  |  |  | - |  |  |  | - |  |  |  | 20.9 |  |  |  | 14.5 |  |  |  | - |  |  |
| >20 |  | - |  |  |  | 13.8 |  |  |  | 15.9 |  |  |  | 33.1 |  |  |  | - |  |  |  | - |  |  |  | - |  |  |  | 15.1 |  |  |  | - |  |  |  | - |  |  |
| Age at menarche, yrs, n(%) |  |  |  |  |  |  |  |  |  |  |  |  |  |  |  |  |  |  |  |  |  |  |  |  |  |  |  |  |  |  |  |  |  |  |  |  |  |  |  |  |
| <12 |  | 17.0 |  | 20.4 |  | 19.5 |  | 25.3 |  | 15.9 |  | 23.2 |  | 17.7 |  | 14.5 |  | 31.2 |  | 26.9 |  | 20.2 |  | 21.6 |  | 19.4 |  | 6.5 |  | 23.7 |  | 26.2 |  | 23.8 |  | 19.2 |  | 19.7 |  | 18.9 |
| 12 - 13 |  | 51.3 |  | 44.3 |  | 59.8 |  | 57.5 |  | 71.0 |  | 50.7 |  | 46.0 |  | 47.6 |  | 58.1 |  | 55.9 |  | 60.5 |  | 58.0 |  | 58.1 |  | 54.8 |  | 55.8 |  | 54.2 |  | 47.7 |  | 55.4 |  | 58.4 |  | 55.6 |
| ≥14 or missing^b^ |  | 31.7 |  | 35.2 |  | 20.7 |  | 17.2 |  | 13.0 |  | 26.1 |  | 36.3 |  | 37.9 |  | 10.8 |  | 17.2 |  | 19.4 |  | 20.4 |  | 22.6 |  | 38.7 |  | 20.5 |  | 19.6 |  | 28.5 |  | 25.4 |  | 22.0 |  | 25.5 |
| Age at first live birth, yrs, n(%) |  |  |  |  |  |  |  |  |  |  |  |  |  |  |  |  |  |  |  |  |  |  |  |  |  |  |  |  |  |  |  |  |  |  |  |  |  |  |  |  |
| <20 or missing^b^ |  | 1.3 |  | 3.9 |  | 16.1 |  | 28.7 |  | 30.4 |  | 18.8 |  | 8.9 |  | 12.9 |  | 2.2 |  | - |  | 2.0 |  | 4.0 |  | 9.7 |  | 9.7 |  | 6.9 |  | 6.9 |  | 3.3 |  | 4.9 |  | 8.1 |  | 7.7 |
| 20 - 24 |  | 16.5 |  | 13.0 |  | 34.5 |  | 39.1 |  | 40.6 |  | 55.1 |  | 41.9 |  | 29.8 |  | 43.0 |  | 44.1 |  | 18.5 |  | 25.2 |  | 29.0 |  | 41.9 |  | 21.5 |  | 21.8 |  | 36.0 |  | 41.1 |  | 17.9 |  | 22.7 |
| 25 - 29 and nulliparous women |  | 50.9 |  | 50.9 |  | 37.9 |  | 25.3 |  | 23.2 |  | 24.6 |  | 41.1 |  | 41.9 |  | 48.4 |  | 52.7 |  | 59.3 |  | 56.8 |  | 45.2 |  | 41.9 |  | 55.0 |  | 55.6 |  | 46.7 |  | 42.0 |  | 53.8 |  | 47.9 |
| ≥ 30 |  | 31.3 |  | 32.2 |  | 11.5 |  | 6.9 |  | 5.8 |  | 1.4 |  | 8.1 |  | 15.3 |  | 6.5 |  | 3.2 |  | 20.2 |  | 14.0 |  | 16.1 |  | 6.5 |  | 16.6 |  | 15.7 |  | 14.0 |  | 12.1 |  | 20.2 |  | 21.7 |
| Number of benign breast biopsies, n(%) |  |  |  |  |  |  |  |  |  |  |  |  |  |  |  |  |  |  |  |  |  |  |  |  |  |  |  |  |  |  |  |  |  |  |  |  |  |  |  |  |
| 0 or missing^b^ |  | 92.2 |  | 96.1 |  | 77.0 |  | 87.4 |  | 84.1 |  | 95.7 |  | 85.5 |  | 87.9 |  | 54.8 |  | 67.7 |  | 75.4 |  | 83.2 |  | 100 |  | 100 |  | 77.7 |  | 84.5 |  | 62.6 |  | 67.4 |  | 63.6 |  | 75.2 |
| ≥1 |  | 7.8 |  | 3.9 |  | 23.0 |  | 12.6 |  | 15.9 |  | 4.3 |  | 14.5 |  | 12.1 |  | 45.2 |  | 32.3 |  | 24.6 |  | 16.8 |  | - |  | - |  | 22.3 |  | 15.5 |  | 37.4 |  | 32.6 |  | 36.4 |  | 24.8 |
| Number of first degree family members with breast cancer, n(%) |  |  |  |  |  |  |  |  |  |  |  |  |  |  |  |  |  |  |  |  |  |  |  |  |  |  |  |  |  |  |  |  |  |  |  |  |  |  |  |  |
| 0 |  | 78.7 |  | 85.2 |  | 86.2 |  | 89.7 |  | 84.1 |  | 95.7 |  | 87.9 |  | 95.2 |  | 91.4 |  | 93.5 |  | 86.7 |  | 89.2 |  | 87.1 |  | 100 |  | 73.8 |  | 81.0 |  | 91.1 |  | 90.6 |  | 1.2 |  | 3.8 |
| 1 ^c^ |  | 19.6 |  | 14.8 |  | 13.8 |  | 10.3 |  | 15.9 |  | 4.3 |  | 10.5 |  | 4.0 |  | 8.6 |  | 6.5 |  | 13.3 |  | 10.8 |  | 12.9 |  | - |  | 24.5 |  | 17.7 |  | 7.9 |  | 9.4 |  | 68.2 |  | 76.6 |
| >1^c^ |  | 1.7 |  | - |  | - |  | - |  | - |  | - |  | 1.6 |  | 0.8 |  | - |  | - |  | - |  | - |  | - |  | - |  | 1.6 |  | 1.2 |  | 0.9 |  | - |  | 30.6 |  | 19.6 |
| BMI, kg/m^2^, n(%) |  |  |  |  |  |  |  |  |  |  |  |  |  |  |  |  |  |  |  |  |  |  |  |  |  |  |  |  |  |  |  |  |  |  |  |  |  |  |  |  |
| <25 |  | 61.8 |  | 56.1 |  | 56.3 |  | 52.9 |  | 53.6 |  | 60.9 |  | 63.7 |  | 62.1 |  | 63.4 |  | 64.8 |  | 65.3 |  | 59.6 |  | 46.7 |  | 50.0 |  | 67.5 |  | 70.1 |  | 62.9 |  | 59.6 |  | 49.7 |  | 43.7 |
| 25 - 29 |  | 24.0 |  | 33.6 |  | 28.7 |  | 21.8 |  | 21.7 |  | 18.8 |  | 29.0 |  | 27.4 |  | 26.9 |  | 20.9 |  | 21.8 |  | 23.6 |  | 33.3 |  | 30.0 |  | 21.5 |  | 20.0 |  | 25.8 |  | 27.8 |  | 28.3 |  | 28.0 |
| ≥30 |  | 14.2 |  | 10.3 |  | 14.9 |  | 25.3 |  | 24.6 |  | 20.3 |  | 7.3 |  | 10.5 |  | 9.7 |  | 14.3 |  | 12.9 |  | 16.8 |  | 20.0 |  | 20.0 |  | 11.0 |  | 9.9 |  | 11.3 |  | 12.6 |  | 22.0 |  | 28.3 |
| AMH cohort-specific quartiles, % |  |  |  |  |  |  |  |  |  |  |  |  |  |  |  |  |  |  |  |  |  |  |  |  |  |  |  |  |  |  |  |  |  |  |  |  |  |  |  |  |
| Q1 |  | 27.0 |  | 25.2 |  | 26.4 |  | 25.3 |  | 20.3 |  | 26.1 |  | 22.6 |  | 25.0 |  | 10.8 |  | 25.8 |  | 20.6 |  | 25.2 |  | 9.7 |  | 25.8 |  | 22.9 |  | 25.8 |  | 12.6 |  | 25.0 |  | 19.7 |  | 25.2 |
| Q2 |  | 25.2 |  | 24.8 |  | 26.4 |  | 25.3 |  | 17.4 |  | 24.6 |  | 27.4 |  | 25.0 |  | 30.1 |  | 24.7 |  | 20.2 |  | 24.8 |  | 29.0 |  | 25.8 |  | 25.6 |  | 24.4 |  | 29.0 |  | 25.0 |  | 24.3 |  | 24.8 |
| Q3 |  | 23.0 |  | 24.8 |  | 19.5 |  | 24.1 |  | 30.4 |  | 24.6 |  | 21.0 |  | 25.0 |  | 29.0 |  | 24.7 |  | 28.2 |  | 24.8 |  | 32.3 |  | 22.6 |  | 25.6 |  | 24.8 |  | 28.0 |  | 25.0 |  | 24.9 |  | 24.8 |
| Q4 |  | 24.8 |  | 25.2 |  | 27.6 |  | 25.3 |  | 31.9 |  | 24.6 |  | 29.0 |  | 25.0 |  | 30.1 |  | 24.7 |  | 31.0 |  | 25.2 |  | 29.0 |  | 25.8 |  | 26.0 |  | 25.0 |  | 30.4 |  | 25.0 |  | 31.2 |  | 25.2 |
| Testosterone cohort-specific quartiles, % |  |  |  |  |  |  |  |  |  |  |  |  |  |  |  |  |  |  |  |  |  |  |  |  |  |  |  |  |  |  |  |  |  |  |  |  |  |  |  |  |
| Q1 |  | 29.1 |  | 27.0 |  | 27.6 |  | 28.7 |  | 15.9 |  | 26.1 |  | 21.8 |  | 27.4 |  | 36.6 |  | 33.3 |  | 26.6 |  | 28.4 |  | 6.5 |  | 25.8 |  | 22.7 |  | 25.4 |  | 25.2 |  | 28.6 |  | 15.0 |  | 25.2 |
| Q2 |  | 23.5 |  | 23.5 |  | 18.4 |  | 24.1 |  | 24.6 |  | 26.1 |  | 18.5 |  | 26.6 |  | 18.3 |  | 18.3 |  | 21.0 |  | 22.0 |  | 35.5 |  | 29.0 |  | 26.0 |  | 27.0 |  | 20.1 |  | 23.2 |  | 30.6 |  | 24.8 |
| Q3 |  | 26.5 |  | 24.8 |  | 18.4 |  | 25.3 |  | 13.0 |  | 23.2 |  | 37.9 |  | 21.0 |  | 30.1 |  | 25.8 |  | 22.6 |  | 26.8 |  | 29.0 |  | 22.6 |  | 25.6 |  | 23.8 |  | 25.2 |  | 23.2 |  | 26.6 |  | 24.8 |
| Q4 |  | 20.9 |  | 24.8 |  | 35.6 |  | 21.8 |  | 46.4 |  | 24.6 |  | 21.8 |  | 25.0 |  | 15.1 |  | 22.6 |  | 29.8 |  | 22.8 |  | 29.0 |  | 22.6 |  | 25.8 |  | 23.8 |  | 29.4 |  | 25.0 |  | 27.7 |  | 25.2 |
| BCRAT 5-year risk score^d^, % |  |  |  |  |  |  |  |  |  |  |  |  |  |  |  |  |  |  |  |  |  |  |  |  |  |  |  |  |  |  |  |  |  |  |  |  |  |  |  |  |
| <0.6% |  | 12.6 |  | 13.0 |  | 28.7 |  | 33.3 |  | 31.9 |  | 31.9 |  | 41.9 |  | 50.0 |  | - |  | - |  | 15.7 |  | 18.8 |  | 3.2 |  | 9.7 |  | 17.4 |  | 19.4 |  | 17.8 |  | 17.9 |  | 2.3 |  | 1.0 |
| 0.6 – 0.99% |  | 42.6 |  | 50.9 |  | 37.9 |  | 41.4 |  | 42.0 |  | 59.4 |  | 42.7 |  | 32.3 |  | 44.1 |  | 48.4 |  | 47.6 |  | 50.4 |  | 54.8 |  | 67.7 |  | 38.9 |  | 43.3 |  | 45.3 |  | 45.5 |  | 0.6 |  | 7.7 |
| 1 - 1.66% |  | 33.5 |  | 31.3 |  | 30.0 |  | 20.7 |  | 17.4 |  | 8.7 |  | 12.9 |  | 13.7 |  | 47.3 |  | 46.2 |  | 28.2 |  | 24.4 |  | 32.3 |  | 22.6 |  | 31.4 |  | 28.2 |  | 29.4 |  | 30.8 |  | 30.1 |  | 29.4 |
| 1.67 - 1.99% |  | 8.3 |  | 4.3 |  | 2.3 |  | 3.4 |  | 2.9 |  | - |  | - |  | 3.2 |  | 3.2 |  | 2.2 |  | 4.4 |  | 4.0 |  | 6.5 |  | - |  | 6.9 |  | 4.6 |  | 4.2 |  | 3.1 |  | 16.2 |  | 24.8 |
| 2 - 2.99% |  | 2.6 |  | 0.4 |  | 1.1 |  | 1.1 |  | 5.8 |  | - |  | 1.6 |  | 0.8 |  | 5.4 |  | 3.2 |  | 4.0 |  | 2.4 |  | 3.2 |  | - |  | 4.9 |  | 4.2 |  | 2.8 |  | 2.7 |  | 32.4 |  | 26.6 |
| ≥3% |  | 0.4 |  | - |  | - |  | - |  | - |  | - |  | 0.8 |  | - |  | - |  | - |  | - |  | - |  | - |  | - |  | 0.4 |  | 0.2 |  | 0.5 |  | - |  | 18.5 |  | 10.5 |
| Estrogen receptor status, % |  |  |  |  |  |  |  |  |  |  |  |  |  |  |  |  |  |  |  |  |  |  |  |  |  |  |  |  |  |  |  |  |  |  |  |  |  |  |  |  |
| ER-positive |  | 88.8 |  |  |  | 78.0 |  |  |  | - |  |  |  | 78.5 |  |  |  | 78.6 |  |  |  | 82.2 |  |  |  | 75.9 |  |  |  | 76.8 |  |  |  | 71.8 |  |  |  | 83.5 |  |  |
| ER-negative |  | 11.2 |  |  |  | 22.0 |  |  |  | - |  |  |  | 21.5 |  |  |  | 21.4 |  |  |  | 17.8 |  |  |  | 24.1 |  |  |  | 23.2 |  |  |  | 28.2 |  |  |  | 16.5 |  |  |
| Missing |  | 6.5 |  |  |  | 5.7 |  |  |  | 100 |  |  |  | 47.6 |  |  |  | 9.7 |  |  |  | 9.4 |  |  |  | 6.5 |  |  |  | 26.6 |  |  |  | 5.6 |  |  |  | 5.2 |  |  |

Note: Cases and controls were matched 1:1 for all cohorts except for Sister Study which matched 1:2. There were 11 postmenopausal case-control matched sets with 1:2 matching in other cohorts (NHS – 2 sets, NYUWHS – 3 sets, ORDET – 6 sets) from a previous study of testosterone.

^a^ All cases had age at blood donation ≤50, though for 24 sets, matched controls ages were ≥51.2 years at blood donation.

^b^ Missing data were recoded as the lowest risk category by Gail Model 2.

^c^ The number of first degree family members with breast cancer was coded as 0 (for no relatives), 1 relative, or > 1 relative. For cohorts that collected family history as a no/yes variable, “yes” answers were assigned to the intermediate category (1 relative).

^d^Calculated using the following variables as they are coded above in Table 1: race, age at menarche, age at first live birth, number of breast biopsies, number of first degree family members with breast cancer, and history of atypical hyperplasia (which was missing for all cohorts and set to “no” for all women, i.e., the lowest risk category was assigned for this variable). Gail model 2 rates and parameters were used as described in [16].

Table S4: Descriptive characteristics of invasive plus in situ breast cancer cases and matched controls, by cohort

|  | BGS | | | | Clue II | | | | CSB | | | | | Guernsey | | | | | | NHS | | | | | NHS II | | | | | | NSMSC | | | | | NYUWHS | | | | | | ORDET | | | | | | | Sister | | | | | |
| --- | --- | --- | --- | --- | --- | --- | --- | --- | --- | --- | --- | --- | --- | --- | --- | --- | --- | --- | --- | --- | --- | --- | --- | --- | --- | --- | --- | --- | --- | --- | --- | --- | --- | --- | --- | --- | --- | --- | --- | --- | --- | --- | --- | --- | --- | --- | --- | --- | --- | --- | --- | --- | --- | --- |
|  | Cases | | Controls | | Cases | | Controls | | Cases | | | Controls | | Cases | | | Controls | | | Cases | | | Controls | | Cases | | | | Controls | | Cases | | Controls | | | Cases | | | Controls | | | Cases | | | Controls | | | | Cases | | Controls | | | |
|  | 268 | | 268 | | 98 | | 98 | | 77 | | | 77 | | 148 | | | 148 | | | 118 | | | 118 | | 356 | | | | 358 | | 31 | | 31 | | | 658 | | | 661 | | | 231 | | | 241 | | | | 247 | | 416 | | | |
| Age at blood donation, yrs, n (%) |  |  |  |  |  |  |  |  |  |  |  | |  | |  |  |  |  |  | |  |  | |  | |  |  |  | |  |  |  | |  |  | |  |  | |  |  | |  |  | |  |  |  | |  | |  |  |  |
| 35 - 40 |  | 16.8 |  | 19.0 |  | 33.7 |  | 31.6 |  | 18.2 |  | | 18.2 | |  | 44.6 |  | 48.0 |  | | - |  | | - | |  | 24.7 |  | | 22.1 |  | 3.3 | |  | 3.2 | |  | 24.0 | |  | 17.9 | |  | 26.4 | |  | 24.1 |  | | 8.9 | |  | 8.4 |  |
| 41 - 45 |  | 42.2 |  | 41.8 |  | 34.7 |  | 33.7 |  | 44.2 |  | | 45.5 | |  | 35.1 |  | 33.1 |  | | 30.5 |  | | 28.8 | |  | 43.5 |  | | 49.4 |  | 19.4 | |  | 22.6 | |  | 38.8 | |  | 36.0 | |  | 38.1 | |  | 35.3 |  | | 30.0 | |  | 29.8 |  |
| 46 - 50 ^a^ |  | 41.0 |  | 39.2 |  | 31.6 |  | 34.7 |  | 36.6 |  | | 36.4 | |  | 18.2 |  | 18.9 |  | | 69.5 |  | | 68.6 | |  | 31.7 |  | | 28.5 |  | 77.4 | |  | 74.2 | |  | 37.2 | |  | 38.7 | |  | 35.5 | |  | 40.6 |  | | 61.1 | |  | 60.8 |  |
| Race/ethnicity, n (%) |  |  |  |  |  |  |  |  |  |  |  | |  | |  |  |  |  |  | |  |  | |  | |  |  |  | |  |  |  | |  |  | |  |  | |  |  | |  |  | |  |  |  | |  | |  |  |  |
| White |  | 99.6 |  | 99.6 |  | 100 |  | 100 |  | 98.7 |  | | 100 | |  | 100 |  | 100 |  | | 98.3 |  | | 99.2 | |  | 97.5 |  | | 97.8 |  | 100 | |  | 100 | |  | 72.2 | |  | 67.3 | |  | 100 | |  | 100 |  | | 84.2 | |  | 88.5 |  |
| Black/African American |  | 0.4 |  | 0.4 |  | - |  | - |  | 1.3 |  | | - | |  | - |  | - |  | | 0.8 |  | | 0.8 | |  | 1.4 |  | | 1.4 |  | - | |  | - | |  | 11.2 | |  | 10.7 | |  | - | |  | - |  | | 7.3 | |  | 7.0 |  |
| Other or missing |  | - |  | - |  | - |  | - |  | - |  | | - | |  | - |  | - |  | | 0.8 |  | |  | |  | 1.1 |  | | 0.8 |  | - | |  | - | |  | 16.6 | |  | 21.9 | |  | - | |  | - |  | | 8.5 | |  | 4.6 |  |
| Age at diagnosis, yrs, n (%) |  |  |  |  |  |  |  |  |  |  |  | |  | |  |  |  |  |  | |  |  | |  | |  |  |  | |  |  |  | |  |  | |  |  | |  |  | |  |  | |  |  |  | |  | |  |  |  |
| 35 - 45 |  | 29.9 |  |  |  | 9.2 |  |  |  | 7.8 |  | |  | |  | 9.5 |  |  |  | | 0.8 |  | |  | |  | 23.0 |  | |  |  | 9.7 | |  |  | |  | 6.8 | |  |  | |  | 10.8 | |  |  |  | | 18.6 | |  |  |  |
| 46 - 50 |  | 47.0 |  |  |  | 17.3 |  |  |  | 10.4 |  | |  | |  | 12.8 |  |  |  | | 32.2 |  | |  | |  | 41.0 |  | |  |  | 25.8 | |  |  | |  | 16.3 | |  |  | |  | 25.5 | |  |  |  | | 50.2 | |  |  |  |
| 51 - 55 |  | 22.8 |  |  |  | 26.5 |  |  |  | 16.9 |  | |  | |  | 18.9 |  |  |  | | 48.3 |  | |  | |  | 32.9 |  | |  |  | 45.2 | |  |  | |  | 22.3 | |  |  | |  | 31.6 | |  |  |  | | 31.2 | |  |  |  |
| 56 - 60 |  | 0.4 |  |  |  | 27.6 |  |  |  | 22.1 |  | |  | |  | 23.0 |  |  |  | | 18.6 |  | |  | |  | 2.8 |  | |  |  | 9.7 | |  |  | |  | 24.6 | |  |  | |  | 23.4 | |  |  |  | | - | |  |  |  |
| 61 - 65 |  | - |  |  |  | 15.3 |  |  |  | 26.0 |  | |  | |  | 14.2 |  |  |  | | - |  | |  | |  | 0.3 |  | |  |  | 9.7 | |  |  | |  | 16.9 | |  |  | |  | 7.4 | |  |  |  | | - | |  |  |  |
| >65 |  | - |  |  |  | 4.1 |  |  |  | 16.9 |  | |  | |  | 21.6 |  |  |  | | - |  | |  | |  | - |  | |  |  | - | |  |  | |  | 13.1 | |  |  | |  | 1.3 | |  |  |  | | - | |  |  |  |
| Lag time between blood donation and diagnosis, yrs, n (%) |  |  |  |  |  |  |  |  |  |  |  | |  | |  |  |  |  |  | |  |  | |  | |  |  |  | |  |  |  | |  |  | |  |  | |  |  | |  |  | |  |  |  | |  | |  |  |  |
| 0 - 2 |  | 32.8 |  |  |  | 9.2 |  |  |  | 9.1 |  | |  | |  | 3.4 |  |  |  | | 12.7 |  | |  | |  | 18.8 |  | |  |  | 25.8 | |  |  | |  | 3.8 | |  |  | |  | 9.5 | |  |  |  | | 38.1 | |  |  |  |
| 3 - 5 |  | 51.1 |  |  |  | 7.1 |  |  |  | 6.5 |  | |  | |  | 7.4 |  |  |  | | 40.6 |  | |  | |  | 31.2 |  | |  |  | 25.8 | |  |  | |  | 9.4 | |  |  | |  | 12.6 | |  |  |  | | 47.4 | |  |  |  |
| 6 - 10 |  | 16.0 |  |  |  | 28.6 |  |  |  | 10.4 |  | |  | |  | 13.5 |  |  |  | | 35.6 |  | |  | |  | 40.2 |  | |  |  | 38.7 | |  |  | |  | 23.3 | |  |  | |  | 30.7 | |  |  |  | | 14.6 | |  |  |  |
| 11 - 15 |  | - |  |  |  | 21.4 |  |  |  | 23.4 |  | |  | |  | 18.2 |  |  |  | | 11.0 |  | |  | |  | 9.8 |  | |  |  | 9.7 | |  |  | |  | 25.7 | |  |  | |  | 33.8 | |  |  |  | | - | |  |  |  |
| 16 - 20 |  | - |  |  |  | 20.4 |  |  |  | 35.1 |  | |  | |  | 22.9 |  |  |  | | - |  | |  | |  | - |  | |  |  | - | |  |  | |  | 22.5 | |  |  | |  | 13.4 | |  |  |  | | - | |  |  |  |
| >20 |  | - |  |  |  | 13.3 |  |  |  | 15.6 |  | |  | |  | 34.5 |  |  |  | | - |  | |  | |  | - |  | |  |  | - | |  |  | |  | 15.4 | |  |  | |  | - | |  |  |  | | - | |  |  |  |
| Age at menarche, yrs, n(%) |  |  |  |  |  |  |  |  |  |  |  | |  | |  |  |  |  |  | |  |  | |  | |  |  |  | |  |  |  | |  |  | |  |  | |  |  | |  |  | |  |  |  | |  | |  |  |  |
| <12 |  | 18.7 |  | 19.4 |  | 19.4 |  | 25.5 |  | 18.2 |  | | 23.4 | |  | 17.6 |  | 16.2 |  | | 30.5 |  | | 25.4 | |  | 20.2 |  | | 22.1 |  | 19.4 | |  | 6.5 | |  | 25.8 | |  | 25.4 | |  | 24.2 | |  | 19.9 |  | | 18.2 | |  | 20.0 |  |
| 12 - 13 |  | 50.7 |  | 45.9 |  | 59.2 |  | 57.1 |  | 70.1 |  | | 53.2 | |  | 45.9 |  | 49.3 |  | | 59.3 |  | | 59.3 | |  | 59.3 |  | | 59.8 |  | 58.1 | |  | 54.8 | |  | 55.2 | |  | 54.9 | |  | 48.1 | |  | 53.9 |  | | 60.7 | |  | 54.1 |  |
| ≥14 or missing^b^ |  | 30.6 |  | 34.7 |  | 21.4 |  | 17.3 |  | 11.7 |  | | 23.4 | |  | 36.5 |  | 34.5 |  | | 10.2 |  | | 15.3 | |  | 20.5 |  | | 18.2 |  | 22.6 | |  | 38.7 | |  | 19.0 | |  | 19.7 | |  | 27.7 | |  | 26.1 |  | | 21.1 | |  | 26.0 |  |
| Age at first live birth, yrs, n(%) |  |  |  |  |  |  |  |  |  |  |  | |  | |  |  |  |  |  | |  |  | |  | |  |  |  | |  |  |  | |  |  | |  |  | |  |  | |  |  | |  |  |  | |  | |  |  |  |
| <20 or missing^b^ |  | 1.5 |  | 3.7 |  | 14.3 |  | 25.5 |  | 29.9 |  | | 19.5 | |  | 9.5 |  | 13.5 |  | | 1.7 |  | | - | |  | 3.4 |  | | 5.0 |  | 9.7 | |  | 9.7 | |  | 5.8 | |  | 6.8 | |  | 3.5 | |  | 5.0 |  | | 7.3 | |  | 8.7 |  |
| 20 - 24 |  | 16.8 |  | 15.3 |  | 35.7 |  | 38.8 |  | 41.6 |  | | 50.6 | |  | 40.5 |  | 30.4 |  | | 44.1 |  | | 45.8 | |  | 17.1 |  | | 24.3 |  | 29.0 | |  | 41.9 | |  | 20.4 | |  | 22.4 | |  | 35.5 | |  | 41.9 |  | | 19.0 | |  | 21.6 |  |
| 25 - 29 and nulliparous women |  | 51.1 |  | 49.3 |  | 37.8 |  | 27.6 |  | 22.1 |  | | 28.6 | |  | 40.5 |  | 40.5 |  | | 45.8 |  | | 50.8 | |  | 60.1 |  | | 56.1 |  | 45.2 | |  | 41.9 | |  | 58.4 | |  | 56.0 | |  | 46.8 | |  | 41.1 |  | | 55.5 | |  | 47.4 |  |
| ≥ 30 |  | 30.6 |  | 31.7 |  | 12.2 |  | 8.2 |  | 6.5 |  | | 1.3 | |  | 9.5 |  | 15.5 |  | | 8.5 |  | | 3.4 | |  | 19.4 |  | | 14.5 |  | 16.1 | |  | 6.5 | |  | 15.5 | |  | 14.8 | |  | 14.3 | |  | 12.0 |  | | 18.2 | |  | 22.4 |  |
| Number of benign breast biopsies, n(%) |  |  |  |  |  |  |  |  |  |  |  | |  | |  |  |  |  |  | |  |  | |  | |  |  |  | |  |  |  | |  |  | |  |  | |  |  | |  |  | |  |  |  | |  | |  |  |  |
| 0 or missing^b^ |  | 90.7 |  | 95.5 |  | 77.6 |  | 87.8 |  | 84.4 |  | | 96.1 | |  | 84.5 |  | 89.2 |  | | 55.1 |  | | 67.8 | |  | 76.7 |  | | 82.1 |  | 100 | |  | 100 | |  | 77.4 | |  | 84.9 | |  | 61.5 | |  | 68.1 |  | | 63.6 | |  | 76.4 |  |
| ≥1 |  | 9.3 |  | 4.5 |  | 22.4 |  | 12.2 |  | 15.6 |  | | 3.9 | |  | 15.5 |  | 10.8 |  | | 44.9 |  | | 32.2 | |  | 23.3 |  | | 17.9 |  | - | |  | - | |  | 22.6 | |  | 15.1 | |  | 38.5 | |  | 32.0 |  | | 36.4 | |  | 23.6 |  |
| Number of first degree family members with breast cancer, n(%) |  |  |  |  |  |  |  |  |  |  |  | |  | |  |  |  |  |  | |  |  | |  | |  |  |  | |  |  |  | |  |  | |  |  | |  |  | |  |  | |  |  |  | |  | |  |  |  |
| 0 |  | 77.6 |  | 85.1 |  | 85.7 |  | 89.8 |  | 80.5 |  | | 96.1 | |  | 87.2 |  | 95.3 |  | | 88.1 |  | | 94.1 | |  | 83.7 |  | | 89.4 |  | 87.1 | |  | 100 | |  | 74.5 | |  | 81.5 | |  | 90.5 | |  | 91.3 |  | | 0.8 | |  | 3.1 |  |
| 1^c^ |  | 19.8 |  | 14.9 |  | 14.3 |  | 10.2 |  | 19.5 |  | | 3.9 | |  | 11.5 |  | 3.4 |  | | 11.9 |  | | 5.9 | |  | 16.3 |  | | 10.6 |  | 12.9 | |  | - | |  | 24.3 | |  | 17.2 | |  | 8.7 | |  | 8.7 |  | | 66.8 | |  | 76.2 |  |
| >1^c^ |  | 2.6 |  | - |  | - |  | - |  | - |  | | - | |  | 1.4 |  | 1.4 |  | | - |  | | - | |  | - |  | | - |  | - | |  | - | |  | 1.2 | |  | 1.2 | |  | 0.9 | |  | - |  | | 32.4 | |  | 20.7 |  |
| BMI, kg/m^2^, n(%) |  |  |  |  |  |  |  |  |  |  |  | |  | |  |  |  |  |  | |  |  | |  | |  |  |  | |  |  |  | |  |  | |  |  | |  |  | |  |  | |  |  |  | |  | |  |  |  |
| <25 |  | 62.2 |  | 58.5 |  | 58.2 |  | 52.0 |  | 54.5 |  | | 61.0 | |  | 63.5 |  | 62.8 |  | | 63.6 |  | | 65.5 | |  | 66.0 |  | | 61.1 |  | 46.7 | |  | 50.0 | |  | 69.6 | |  | 69.5 | |  | 63.5 | |  | 58.6 |  | | 50.6 | |  | 42.3 |  |
| 25 - 29 |  | 24.4 |  | 30.4 |  | 27.6 |  | 21.4 |  | 20.8 |  | | 20.8 | |  | 29.1 |  | 26.4 |  | | 26.3 |  | | 21.6 | |  | 21.9 |  | | 23.2 |  | 33.3 | |  | 30.0 | |  | 20.4 | |  | 20.5 | |  | 25.2 | |  | 28.5 |  | | 30.4 | |  | 30.3 |  |
| ≥30 |  | 13.4 |  | 11.2 |  | 14.3 |  | 26.5 |  | 24.7 |  | | 18.2 | |  | 7.4 |  | 10.8 |  | | 10.2 |  | | 12.9 | |  | 12.1 |  | | 15.7 |  | 20.0 | |  | 20.0 | |  | 10.0 | |  | 10.0 | |  | 11.3 | |  | 13.0 |  | | 19.0 | |  | 27.4 |  |
| AMH cohort-specific quartiles, % |  |  |  |  |  |  |  |  |  |  |  | |  | |  |  |  |  |  | |  |  | |  | |  |  |  | |  |  |  | |  |  | |  |  | |  |  | |  |  | |  |  |  | |  | |  |  |  |
| Q1 |  | 26.9 |  | 25.0 |  | 25.5 |  | 25.5 |  | 20.8 |  | | 26.0 | |  | 23.6 |  | 25.0 |  | | 12.7 |  | | 25.4 | |  | 19.9 |  | | 25.1 |  | 9.7 | |  | 25.8 | |  | 22.8 | |  | 25.6 | |  | 13.9 | |  | 25.3 |  | | 20.6 | |  | 25.0 |  |
| Q2 |  | 25.4 |  | 25.0 |  | 21.4 |  | 24.5 |  | 16.9 |  | | 24.7 | |  | 26.4 |  | 25.0 |  | | 25.4 |  | | 24.6 | |  | 18.0 |  | | 24.9 |  | 29.0 | |  | 25.8 | |  | 25.8 | |  | 24.8 | |  | 28.1 | |  | 24.9 |  | | 24.7 | |  | 25.0 |  |
| Q3 |  | 22.4 |  | 25.0 |  | 21.4 |  | 24.5 |  | 23.4 |  | | 24.7 | |  | 24.3 |  | 25.0 |  | | 28.8 |  | | 24.6 | |  | 33.1 |  | | 24.9 |  | 32.3 | |  | 22.6 | |  | 23.7 | |  | 24.8 | |  | 26.8 | |  | 24.9 |  | | 25.5 | |  | 25.0 |  |
| Q4 |  | 25.4 |  | 25.0 |  | 31.6 |  | 25.5 |  | 39.0 |  | | 24.7 | |  | 25.7 |  | 25.0 |  | | 33.1 |  | | 25.4 | |  | 28.9 |  | | 25.1 |  | 29.0 | |  | 25.8 | |  | 27.7 | |  | 24.8 | |  | 31.2 | |  | 24.9 |  | | 29.2 | |  | 25.0 |  |
| Testosterone cohort-specific quartiles, % |  |  |  |  |  |  |  |  |  |  |  | |  | |  |  |  |  |  | |  |  | |  | |  |  |  | |  |  |  | |  |  | |  |  | |  |  | |  |  | |  |  |  | |  | |  |  |  |
| Q1 |  | 27.2 |  | 25.7 |  | 26.5 |  | 27.6 |  | 18.2 |  | | 26.0 | |  | 20.3 |  | 26.4 |  | | 35.6 |  | | 30.5 | |  | 26.1 |  | | 27.7 |  | 6.5 | |  | 25.8 | |  | 26.4 | |  | 28.1 | |  | 25.1 | |  | 28.2 |  | | 20.2 | |  | 25.0 |  |
| Q2 |  | 26.9 |  | 24.3 |  | 17.3 |  | 26.5 |  | 22.1 |  | | 24.7 | |  | 18.9 |  | 26.4 |  | | 22.0 |  | | 28.8 | |  | 23.3 |  | | 26.0 |  | 35.5 | |  | 29.0 | |  | 20.7 | |  | 24.1 | |  | 23.4 | |  | 25.3 |  | | 29.6 | |  | 25.0 |  |
| Q3 |  | 23.5 |  | 25.4 |  | 19.4 |  | 23.5 |  | 14.3 |  | | 24.7 | |  | 37.2 |  | 23.0 |  | | 22.0 |  | | 16.1 | |  | 21.9 |  | | 22.1 |  | 29.0 | |  | 22.6 | |  | 27.8 | |  | 23.9 | |  | 22.9 | |  | 22.4 |  | | 25.5 | |  | 25.0 |  |
| Q4 |  | 22.4 |  | 24.6 |  | 36.7 |  | 22.4 |  | 45.5 |  | | 24.7 | |  | 23.6 |  | 24.3 |  | | 20.3 |  | | 24.6 | |  | 28.7 |  | | 24.3 |  | 29.0 | |  | 22.6 | |  | 25.1 | |  | 23.9 | |  | 28.6 | |  | 24.1 |  | | 24.7 | |  | 25.0 |  |
| BCRAT 5-year risk score^d^, % |  |  |  |  |  |  |  |  |  |  |  | |  | |  |  |  |  |  | |  |  | |  | |  |  |  | |  |  |  | |  |  | |  |  | |  |  | |  |  | |  |  |  | |  | |  |  |  |
| <0.6% |  | 11.6 |  | 12.7 |  | 27.6 |  | 32.7 |  | 31.2 |  | | 29.9 | |  | 41.2 |  | 48.6 |  | | - |  | | - | |  | 18.0 |  | | 22.6 |  | 3.2 | |  | 9.7 | |  | 16.4 | |  | 19.7 | |  | 17.7 | |  | 18.3 |  | | 2.0 | |  | 0.7 |  |
| 0.6 - 0.99% |  | 43.3 |  | 50.4 |  | 40.8 |  | 42.9 |  | 40.3 |  | | 61.0 | |  | 41.2 |  | 34.5 |  | | 42.4 |  | | 50.8 | |  | 45.5 |  | | 46.9 |  | 54.8 | |  | 67.7 | |  | 40.0 | |  | 44.5 | |  | 43.7 | |  | 45.6 |  | | 1.2 | |  | 7.5 |  |
| 1 - 1.66% |  | 31.3 |  | 32.1 |  | 27.6 |  | 20.4 |  | 18.2 |  | | 9.1 | |  | 14.2 |  | 13.5 |  | | 45.8 |  | | 44.1 | |  | 27.8 |  | | 25.1 |  | 32.3 | |  | 22.6 | |  | 31.2 | |  | 27.4 | |  | 29.0 | |  | 30.7 |  | | 29.6 | |  | 30.5 |  |
| 1.67 - 1.99% |  | 9.3 |  | 4.1 |  | 2.0 |  | 3.1 |  | 3.9 |  | | - | |  | - |  | 2.7 |  | | 5.1 |  | | 2.5 | |  | 4.8 |  | | 3.4 |  | 6.5 | |  | - | |  | 7.8 | |  | 4.2 | |  | 6.1 | |  | 2.9 |  | | 17.4 | |  | 24.0 |  |
| 2 - 2.99% |  | 3.7 |  | 0.7 |  | 2.0 |  | 1.0 |  | 6.5 |  | | - | |  | 2.7 |  | 0.7 |  | | 6.8 |  | | 2.5 | |  | 3.9 |  | | 2.0 |  | 3.2 | |  | - | |  | 4.4 | |  | 3.9 | |  | 3.0 | |  | 2.5 |  | | 30.0 | |  | 26.2 |  |
| ≥3% |  | 0.7 |  | - |  | - |  | - |  | - |  | | - | |  | 0.7 |  | - |  | | - |  | | - | |  | - |  | | - |  | - | |  | - | |  | 0.3 | |  | 0.3 | |  | 0.4 | |  | - |  | | 19.8 | |  | 11.1 |  |
| Estrogen receptor status, % |  |  |  |  |  |  |  |  |  |  |  | |  | |  |  |  |  |  | |  |  | |  | |  |  |  | |  |  |  | |  |  | |  |  | |  |  | |  |  | |  |  |  | |  | |  |  |  |
| ER-positive |  | 87.2 |  |  |  | 79.3 |  |  |  | - |  | |  | |  | 77.3 |  |  |  | | 78.9 |  | |  | |  | 82.8 |  | |  |  | 75.9 | |  |  | |  | 74.7 | |  |  | |  | 72.5 | |  |  |  | | 83.5 | |  |  |  |
| ER-negative |  | 12.8 |  |  |  | 20.7 |  |  |  | - |  | |  | |  | 22.7 |  |  |  | | 21.1 |  | |  | |  | 27.2 |  | |  |  | 24.1 | |  |  | |  | 25.3 | |  |  | |  | 27.5 | |  |  |  | | 16.5 | |  |  |  |
| Missing |  | 12.3 |  |  |  | 11.2 |  |  |  | 100 |  | |  | |  | 49.3 |  |  |  | | 23.7 |  | |  | |  | 23.3 |  | |  |  | 6.5 | |  |  | |  | 35.7 | |  |  | |  | 5.6 | |  |  |  | | 9.3 | |  |  |  |

Note: Cases and controls were matched 1:1 for all cohorts except for Sister Study which matched 1:2.

^a^ All cases had age at blood donation ≤50. Matched controls ages were up to 51.2 years at blood donation.

^b^ Missing data were recoded as the lowest risk category by Gail Model 2.

^c^ The number of first degree family members with breast cancer was coded as 0 (for no relatives), 1 relative, or > 1 relative. For cohorts that collected family history as a no/yes variable, “yes” answers were assigned to the intermediate category (1 relative).

^d^ Calculated using the following variables as they are coded above in Table 1: race, age at menarche, age at first live birth, number of breast biopsies, number of first degree family members with breast cancer, and history of atypical hyperplasia (which was missing for all cohorts and set to “no” for all women, i.e., the lowest risk category was assigned for this variable). Gail model 2 rates and parameters were used as described in [16].

Table S5: Random effects meta-analysis relative risk estimates, invasive and *in situ*

| Risk factor | RR estimates (95% CI) | | | |
| --- | --- | --- | --- | --- |
|  | Gail | Gail + AMH | Gail + Testosterone | Gail + AMH + Testosterone |
| Age at menarche, yrs |  |  |  |  |
| <12 | 1.03 (0.94,1.13) | 1.04 (0.95,1.15) | 1.02 (0.93,1.12) | 1.04 (0.95,1.14) |
| 12-13 | 1.01 (0.93,1.11) | 1.02 (0.93,1.12) | 1.01 (0.92,1.11) | 1.02 (0.93,1.12) |
| ≥14 | 1.0 (ref) | 1.0 (ref) | 1.0 (ref) | 1.0 (ref) |
| Age at first live birth, yrs |  |  |  |  |
| < 20 | 1.0 (ref) | 1.0 (ref) | 1.0 (ref) | 1.0 (ref) |
| 20-24 | 1.13 (1.03,1.24) | 1.13 (1.03,1.24) | 1.14 (1.03,1.26) | 1.14 (1.03,1.26) |
| 25-29 and nulliparous | 1.28 (1.16,1.41) | 1.28 (1.17,1.41) | 1.29 (1.17,1.43) | 1.29 (1.17,1.43) |
| ≥ 30 | 1.44 (1.31,1.59) | 1.45 (1.32,1.59) | 1.47 (1.33,1.62) | 1.47 (1.33,1.62) |
| Number of benign breast biopsies |  |  |  |  |
| 0 | 1.0 (ref) | 1.0 (ref) | 1.0 (ref) | 1.0 (ref) |
| ≥1 | 1.61 (1.38,1.87) | 1.58 (1.35,1.85) | 1.63 (1.40,1.90) | 1.60 (1.36,1.88) |
| Number of first degree family members with breast cancer^a^ |  |  |  |  |
| 0 | 1.0 (ref) | 1.0 (ref) | 1.0 (ref) | 1.0 (ref) |
| 1 | 1.65 (1.41,1.93) | 1.64 (1.40,1.92) | 1.64 (1.40,1.91) | 1.62 (1.39,1.90) |
| >1 | 2.74 (2.34,3.20) | 2.69 (2.30,3.14) | 2.67 (2.29,3.13) | 2.64 (2.25,3.09) |
| AMH |  |  |  |  |
| Q1 | - | 1.0 (ref) | - | 1.0 (ref) |
| Q2 | - | 1.16 (1.04,1.29) | - | 1.15 (1.04,1.27) |
| Q3 | - | 1.34 (1.21,1.49) | - | 1.32 (1.19,1.46) |
| Q4 | - | 1.56 (1.40,1.73) | - | 1.52 (1.37,1.68) |
| Testosterone |  |  |  |  |
| Q1 | - | - | 1.0 (ref) | 1.0 (ref) |
| Q2 | - | - | 1.07 (1.01,1.13) | 1.06 (1.00,1.12) |
| Q3 | - | - | 1.15 (1.09,1.21) | 1.12 (1.06,1.18) |
| Q4 | - | - | 1.23 (1.16,1.30) | 1.18 (1.12,1.25) |

^a^ The number of first degree family members with breast cancer was coded as 0 (for no relatives), 1 relative, or > 1 relative. For cohorts that collected family history as a no/yes variable, “yes” answers were assigned to the intermediate category (1 relative).

Figure S1: Cohort-specific and random effects meta-analysis relative risk estimates for Gail model variables, AMH and testosterone (invasive cases only)

**AMH (per one quartile increase)**

**Age at menarche (per category decrease)**

**History of breast biopsy (yes/no)**

**First degree relatives with breast cancer (per category increase: 0, 1, >1 affected relatives)**

**Testosterone (per one quartile increase)**

**Age at first full term pregnancy (per category increase)**

Figure S2: Cohort-specific and random effects meta-analysis relative risk estimates for Gail model variables, AMH and testosterone, invasive and *in situ*

**History of breast biopsy (yes/no)**

**Age at menarche (per category decrease)**

**Age at first full term pregnancy (per category increase)**

**AMH (per one quartile increase)**

**Testosterone (per one quartile increase)**

**First degree relatives with breast cancer (0, 1, >1 affected relatives)**

Figure S3: Relative risk estimates by age group, invasive cases only

**Testosterone (per one quartile increase)**

**AMH (per one quartile increase)**

**Age at menarche (per category decrease)**

**Age at first full term pregnancy (per category increase)**

**First degree relatives with breast cancer (per category increase: 0, 1, >1 affected relatives)**

**History of breast biopsy (yes/no)**


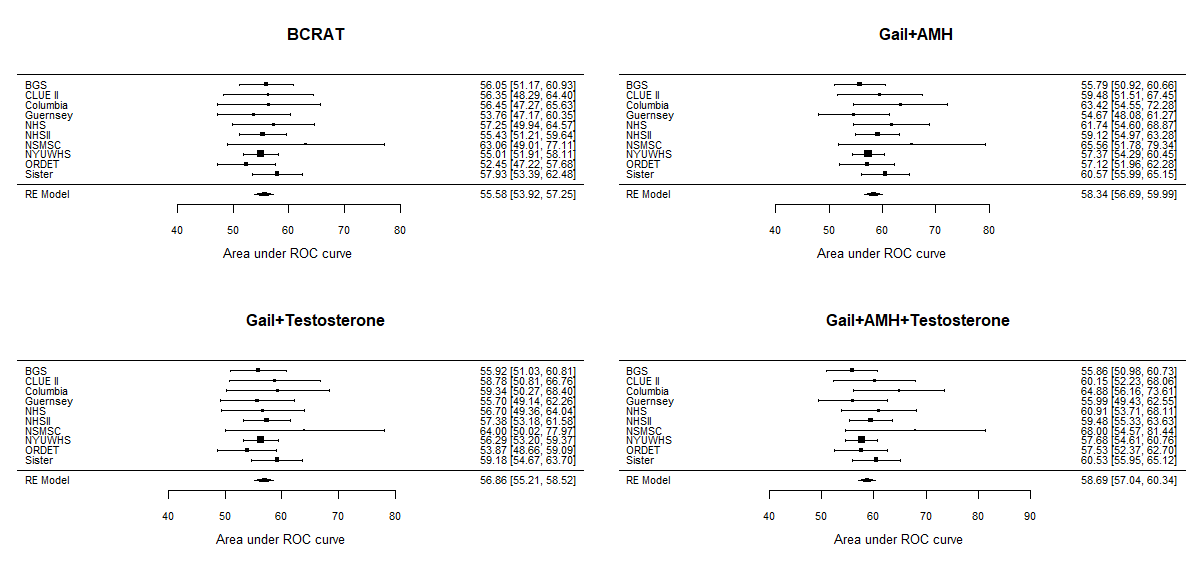
Figure S4: AUCs by cohort 95% confidence intervals, invasive and *in situ*

b

a

b

b

a

^a^  Model as implemented in BCRAT using the BCRAT model variables and regression coefficients.

^b^ Model including Gail model variables and biomarker(s) and using regression coefficients in Supplementary Table 5.

References

1. Dorgan JF, Stanczyk FZ, Kahle LL, Brinton LA: **Prospective case-control study of premenopausal serum estradiol and testosterone levels and breast cancer risk**. *Breast cancer research : BCR* 2010, **12**(6):R98.

2. Schoemaker MJ, Folkerd EJ, Jones ME, Rae M, Allen S, Ashworth A, Dowsett M, Swerdlow AJ: **Combined effects of endogenous sex hormone levels and mammographic density on postmenopausal breast cancer risk: results from the Breakthrough Generations Study**. *Br J Cancer* 2014, **110**(7):1898-1907.

3. Thomas HV, Key TJ, Allen DS, Moore JW, Dowsett M, Fentiman IS, Wang DY: **A prospective study of endogenous serum hormone concentrations and breast cancer risk in premenopausal women on the island of Guernsey**. *Br J Cancer* 1997, **75**(7):1075-1079.

4. Zeleniuch-Jacquotte A, Afanasyeva Y, Kaaks R, Rinaldi S, Scarmo S, Liu M, Arslan AA, Toniolo P, Shore RE, Koenig KL: **Premenopausal serum androgens and breast cancer risk: a nested case-control study**. *Breast cancer research : BCR* 2012, **14**(1):R32.

5. Micheli A, Muti P, Secreto G, Krogh V, Meneghini E, Venturelli E, Sieri S, Pala V, Berrino F: **Endogenous sex hormones and subsequent breast cancer in premenopausal women**. *International journal of cancer Journal international du cancer* 2004, **112**(2):312-318.

6. Nichols HB, Baird DD, Stanczyk FZ, Steiner AZ, Troester MA, Whitworth KW, Sandler DP: **Anti-Mullerian Hormone Concentrations in Premenopausal Women and Breast Cancer Risk**. *Cancer prevention research (Philadelphia, Pa)* 2015.

7. Fortner RT, Eliassen AH, Spiegelman D, Willett WC, Barbieri RL, Hankinson SE: **Premenopausal endogenous steroid hormones and breast cancer risk: results from the Nurses' Health Study II**. *Breast cancer research : BCR* 2013, **15**(2):R19.

8. Ge W, Clendenen TV, Afanasyeva Y, Koenig KL, Agnoli C, Brinton LA, Dorgan JF, Eliassen AH, Falk RT, Hallmans G *et al*: **Circulating anti-Mullerian hormone and breast cancer risk: A study in ten prospective cohorts**. *International journal of cancer Journal international du cancer* 2018, **142**(11):2215-2226.

9. Chen J, Pee D, Ayyagari R, Graubard B, Schairer C, Byrne C, Benichou J, Gail MH: **Projecting absolute invasive breast cancer risk in white women with a model that includes mammographic density**. *Journal of the National Cancer Institute* 2006, **98**(17):1215-1226.

10. Gail MH, Costantino JP, Pee D, Bondy M, Newman L, Selvan M, Anderson GL, Malone KE, Marchbanks PA, McCaskill-Stevens W *et al*: **Projecting individualized absolute invasive breast cancer risk in African American women**. *J Natl Cancer Inst* 2007, **99**(23):1782-1792.

11. Bruzzi P, Green SB, Byar DP, Brinton LA, Schairer C: **Estimating the population attributable risk for multiple risk factors using case-control data**. *Am J Epidemiol* 1985, **122**(5):904-914.

12. **USA: National Center for Health Statistics, Centers for Disease Control and Prevention.** [<https://www.cdc.gov/nchs/data_access/cmf.htm>]

13. **CI5 Cancer Incidence in Five Continents** [<http://ci5.iarc.fr/CI5I-X/Pages/references.aspx>]

14. **World Health Organization, Department of Information, Evidence and Research, mortality database** [<http://www.who.int/healthinfo/mortality_data/en/>]

15. **UK, England and Wales: Office for National Statistics Twentieth Century Mortality Files.** [<https://data.gov.uk/dataset/2548e46b-873e-4668-968c-25d6c155dd73/the-20th-century-mortality-files>]

16. Costantino JP, Gail MH, Pee D, Anderson S, Redmond CK, Benichou J, Wieand HS: **Validation studies for models projecting the risk of invasive and total breast cancer incidence**. *J Natl Cancer Inst* 1999, **91**(18):1541-1548.
